# The Influence of Methyl Groups on the Formation of the Ferroelectric Nematic Phase

**DOI:** 10.1021/acsomega.5c02500

**Published:** 2025-05-27

**Authors:** Ewan Cruickshank, Rebecca Walker, Magdalena M. Majewska, Ewa Gorecka, Damian Pociecha, John M. D. Storey, Corrie T. Imrie

**Affiliations:** † Department of Chemistry, 1019 University of Aberdeen, Old Aberdeen AB24 3UE, U.K.; ‡ Faculty of Chemistry, 49605 University of Warsaw, Zwirki i Wigury 101, Warsaw 02-089, Poland

## Abstract

The synthesis and characterization of 12 ferroelectric nematogens based on the RM734 structural template are reported in the form of two series named *n*ECMe and *n*ECMeF. Within both series, the position of the methyl groups present was varied and all 12 of these compounds exhibited the ferroelectric nematic phase. In general, when modifications were made that decreased the shape anisotropy of the molecule, the value of *T*_NI_ decreased in line with the literature. The behavior of *T*_N_F_N/I_ was, however, much more complex, appearing to be directed by the substituents present, and the transition temperatures did not follow a distinct trend as modifications were made. This is particularly apparent when comparing 2ECMe and 3ECMe which both have a terminal methyl group and a lateral methoxy group: the *T*_N_F_N_ of 3ECMe is 25 °C higher than that of 2ECMe, while their equivalent methoxy-substituted materials have transition temperatures essentially identical to one another. The properties of these materials can be justified, in general, by the model of Madhusudana and are sensitive to the electronic distribution within the compounds.

## Graphical abstract

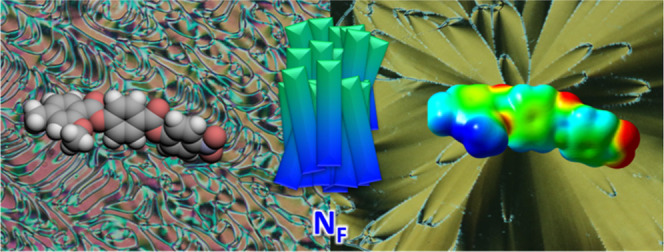

## Introduction

The ferroelectric nematic phase, N_F_, has become one of the key research topics in the field of liquid crystals since its experimental discovery in 2017 ^1,2^ and assignment in 2020,^3^ with an ever growing library of compounds and a wide range of potential applications being identified.^4–6^ The N_F_ phase is a variant of the conventional nematic phase, N, which is the least ordered liquid crystalline phase. In the N phase, the molecules align, in general, in the same direction known as the director, represented by *n*, and the nonpolar nature of the phase means that it possesses inversion symmetry so that *n* = −*n*, Figure 1. On becoming the polar N_F_ phase, this inversion symmetry is broken such that *n* ≠ −*n* and the dipoles therefore align, Figure 1. The ferroelectric nematic phase has attracted significant attention thanks to its properties, which give these materials the possibility of having consequential real-world applications. These properties include ease of alignment,^7,8^ high nonlinear optical activity,^9–11^ and high polarization values;^2,3,12^ however, recent studies have also shown that the N_F_ phase can exhibit other fascinating properties outside of these.^2,3,13–27^

**Figure 1:**
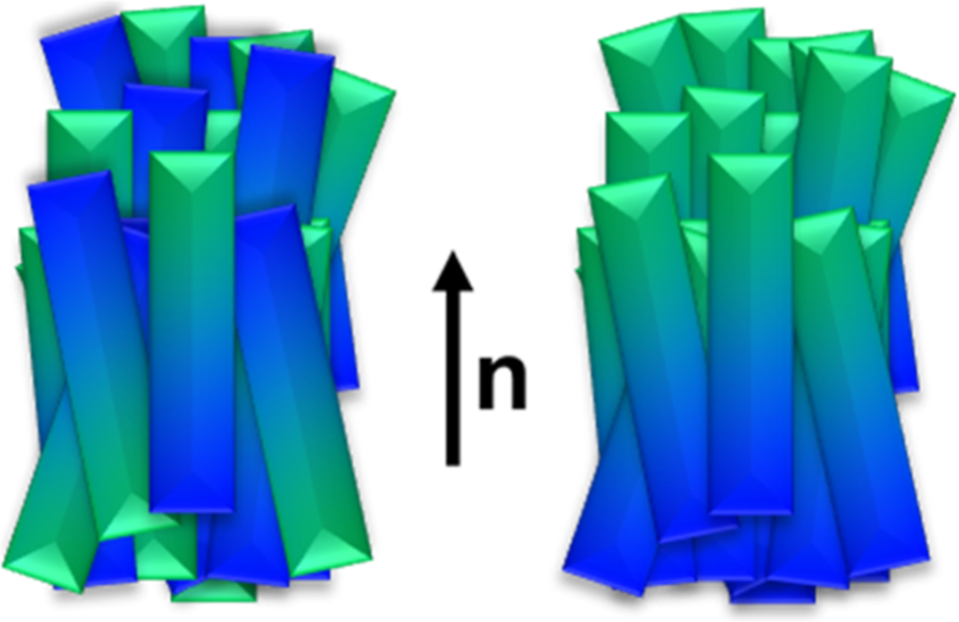
Schematic illustration of (left) the conventional nematic phase and (right) the ferroelectric nematic phase.

To date, there have been around 150 low molar mass compounds which exhibit the phase,^11,24,25,28–42^ and the vast majority of these can be described using the three following structures, namely, RM734,^1,39^ DIO,^2^ and UUQU-4N, Figure 2.^43^ These templates, although having differences in their structures, have similar features: namely, a large longitudinal dipole and some degree of lateral substitution, whether it be fluorination or alkyloxy chains. While there are some exceptions to these templates such as rigid fluorinated molecules,^44–46^ polymers,^36,47,48^ and a hydrogen-bonded ferroelectric nematogen which has a surprisingly small molecular dipole,^49^ these examples are much less common. Indeed, there has been the discovery of a range of other fascinating phases along with the N_F_ phase, such as the antiferroelectric N_X_ phase also called SmZ_A_,^31,38,50,51^ several polar smectic phases,^23,44,52–54^ and even heliconical ferroelectric nematic phases.^55,56^ Clearly, it is therefore critical that the library of compounds be expanded into either new structure spaces or with new molecular functionality to better understand the driving forces behind the stability of the N_F_ and these other new polar phases.

**Figure 2:**
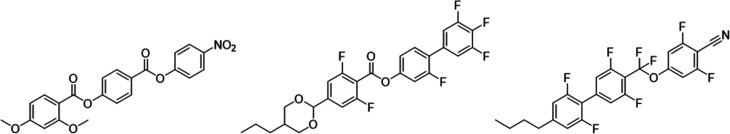
Molecular structure of (left) RM734, (middle) DIO, and (right) UUQU-4N.

This work will focus on materials based on the molecule RM734 which, of the three archetypal molecules, has seen the most extensive examination of its structure. Structural modifications which have been shown to maintain the N_F_ phase have included changing the terminal alkyloxy chain length,^31,39^ the lateral alkyloxy chain length,^11,28,42^ the position of the lateral alkyloxy chain,^25^ the degree of fluorination,^39,41,57^ the nature of the terminal group at either end of the molecule,^29,37,58,59^ the nature of the lateral groups,^32,37^ the inclusion of a biphenyl moiety,^28^ the inclusion of a pyridine moiety,^40^ the number of lateral methoxy groups,^30^ and a combination of such functionalities on the molecule.^37^ Here, we replace the methoxy groups in the terminal ring of RM734 with methyl groups to reduce the electron density within that aromatic ring and therefore hope to stabilize the N_F_ phase in accordance with the model proposed by Madhusudana.^60^ The model describes these molecules using regions of alternating positive and negative charges, with these regions being separated by the ester groups. The electron density present is dictated by the electron-withdrawing or electron-donating nature of the functional groups attached to the aromatic rings. The terminus of the rod with the methoxy group is positively charged, and the nitro terminus is negative, which gives the large longitudinal dipole moment. When the rod-like molecules can exist in close proximity, then the strong dipolar interactions can inhibit the formation of antiparallel structures which are unfavorable for the formation of the N_F_ phase. Specifically, this suggests that by minimizing the amplitude of the charge density wave at either end of the molecule, parallel structures can be favored and the N_F_ phase stabilized, and this in principle can be achieved by using a weaker electron donor such as a methyl group.

We therefore report the phase behavior of 12 ferroelectric nematogens based on RM734 which contain a methyl group or groups in the terminal aromatic ring. Of these compounds, 6 are terminated with a nitrophenol moiety and are known as the *n*ECMe series, while 6 are terminated with a 3-fluoronitrophenol moiety and are known as the *n*ECMeF series, both are summarized in Table 1.

**Table 1:**
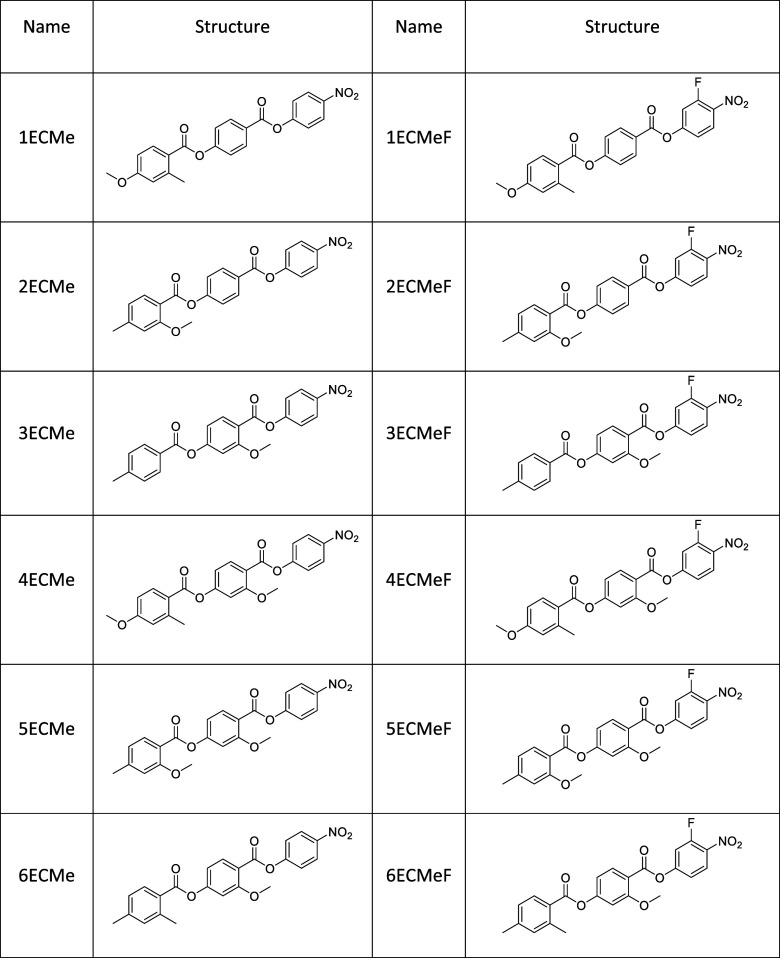
Compound Names and Structures for the Members of the *n*ECMe and *n*ECMeF Series

## Experimental Section

The synthetic route used to prepare 1ECMe, 2ECMe, 1ECMeF, and 2ECMEF is shown in Scheme 1, while 3ECMe-6ECMe and 3ECMeF-6ECMeF are shown in Scheme 2. A detailed description of the preparation of all the intermediates and final products, including full structural characterization, is provided in the Supporting Information.

**Scheme 1:**
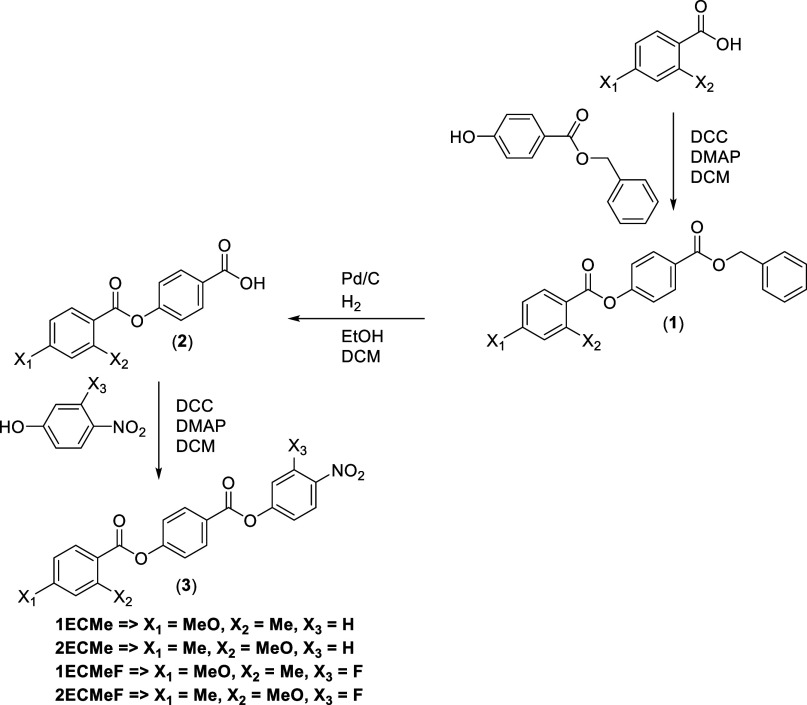
Synthesis of 1ECMe, 2ECMe, 1ECMeF, and 2ECMeF

**Scheme 2:**
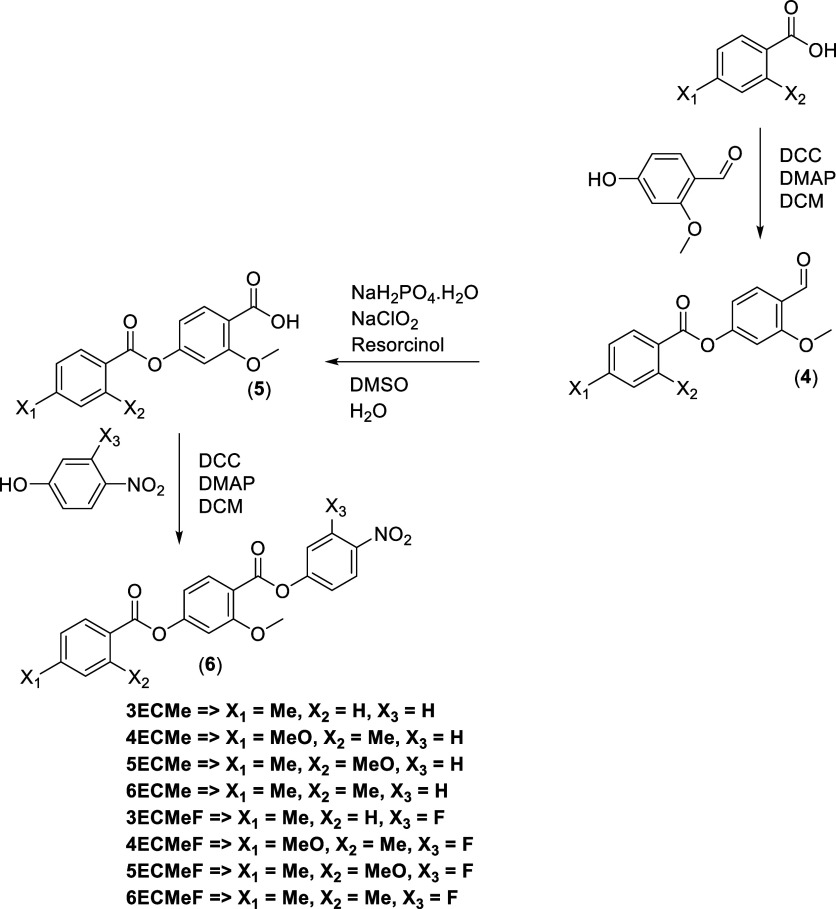
Synthesis of 3ECMe-6ECMe and 3ECMeF-6ECMeF

### Optical Studies

Phase characterization was performed by polarized optical microscopy using a Zeiss AxioImager A2m equipped with a Linkam THMS600 hot stage. Slides treated for homotropic alignment were purchased from AWAT with a thickness of 1.7 μm.

### Differential Scanning Calorimetry

The phase behavior of the materials was studied by differential scanning calorimetry performed using a Mettler Toledo DSC1 or a DSC3 differential scanning calorimeter equipped with TSO 801RO sample robots and calibrated using indium and zinc standards. Heating and cooling rates were 10 K min^–1^, with a 3 min isotherm between either heating or cooling, and all samples were measured under a nitrogen atmosphere. Transition temperatures and associated enthalpy changes were extracted from the heating traces, unless otherwise noted. For each sample, two aliquots were measured, and the data listed are the average of the two sets.

### Molecular Modeling

The geometric parameters of the compounds of interest were obtained using quantum mechanical DFT calculations with Gaussian09 software.^61^ Optimization of the molecular structures was carried out at the B3LYP/6-31G­(d) level. Visualizations of electronic surfaces and ball-and-stick models were generated from the optimized geometries using the GaussView 5 software. The electronic surfaces were found with the cubegen utility in GaussView by generating a total density cube using a SCF density matrix and course grid, which were overlaid by an ESP surface map. Visualizations of the space-filling models were produced postoptimization using the QuteMol package.^62^

### Dielectric Spectroscopy

The complex dielectric permittivity, ε*, was studied using a Solartron 1260 impedance analyzer. Measurements were conducted in the 1 Hz to 1 MHz frequency (*f*) range, with a probe voltage of 20 mV, and it was checked by optical observations that such a voltage is below the Fredericks transition threshold. The material was placed in 9.7 μm-thick glass cells with ITO electrodes and no polymer aligning layers. Lack of a surfactant layer resulted in the random configuration of the director in the LC phases; microscopic observations of optical textures suggested a dominant planar orientation without preferable direction of the long molecular axis. The relaxation frequency, *f*_r_, and dielectric strength of the mode, Δε, were evaluated by fitting the complex dielectric permittivity to the Cole–Cole formula: where ε_∞_ is the high-frequency dielectric constant, α is the distribution parameter of the mode, and δ is the low-frequency conductivity, respectively.

## Results and Discussion

The transitional properties of the *n*ECMe series are reported in Table 2. 2ECMe has been previously reported in the literature, and the transition temperatures here are in good agreement.^32^ 1ECMe, 2ECMe, 3ECMe, and 4ECMe all have the phase sequence of N–N_F_ on cooling from the isotropic phase, with the conventional nematic phase being assigned by the observation of a characteristic schlieren texture with two- and four-brush point defects, Figure 3a. On further cooling into the ferroelectric nematic phase, the defect pattern is replaced by a characteristic banded texture where there are birefringent domains separated by distinct domain walls, Figure 3b. For 5ECMe and 6ECMe, the conventional nematic phase is extinguished, and instead, there is a direct transition between the isotropic and ferroelectric nematic phases, and when a homeotropically aligned cell was used, we again observed a banded texture, Figure 3c. In these cells, we also observed regions with parabolic domain walls as shown in Figure 3d, which are similar to those reported by Kumari *et al*.^63^ The values of the scaled entropy change associated with the N_F_–I transition, Δ*S*_N_F_I_/*R*, listed in Table 2 are similar to those previously reported^25,30,42^ and are much larger than those observed for a transition to the conventional nematic phase. This additional entropic contribution is thought to be associated with ordering of the dipoles in the N_F_ phase.

**Table 2:** Transition Temperatures and Associated Entropy Changes for the *n*ECMe Series^a^

compound	*T*_Cr–_/°C	*T*_N_F_N_/°C *T_N_F_I_/°C	*T*_NI_/°C	Δ*S*_Cr–_/*R*	*Δ*S*_N_F_N_/*R* Δ*S*_N_F_I_/*R*	Δ*S*_NI_/*R*	μ/D
1ECMe	152	^c^71	218	10.7		0.13	9.91
2ECMe	158	^b^128	^b^156	11.6	^b^0.26	^b^0.15	10.8
3ECMe	164	^b^153	171	11.7	^b^0.22	0.31	10.8
4ECMe	153	^b^113	^b^120	11.8	^b^0.40	^b^0.20	11.1
5ECMe	147	*^b^106		13.3	*^b^1.54		12.3
6ECMe	150	*^b^117		12.1	*^b^1.28		10.6

a The calculated dipole moments, μ, are also listed.

b Values extracted from DSC cooling traces.

c Measured using polarized optical microscopy.

**Figure 3:**
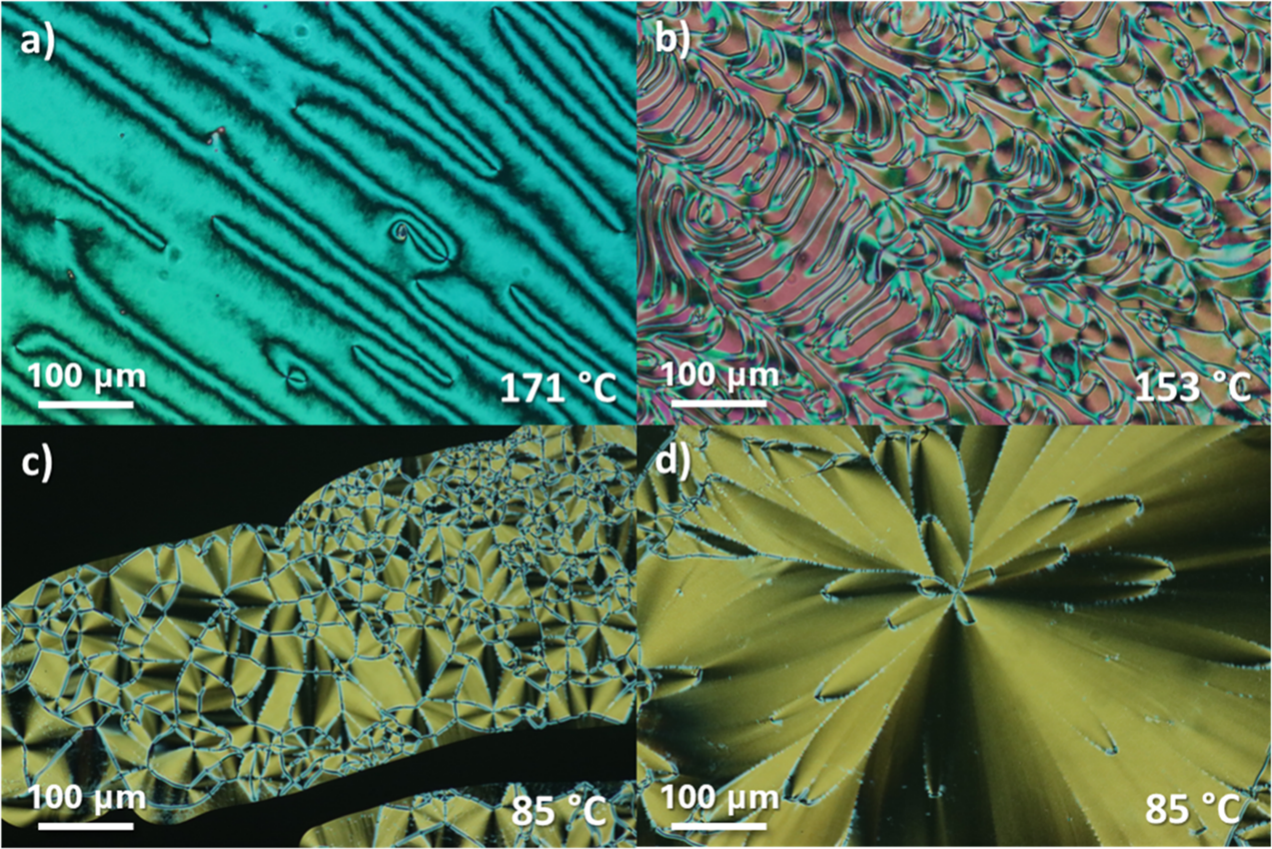
Polarized optical microscope textures for the *n*ECMe series: (a) schlieren texture of the nematic phase and (b) banded texture of the ferroelectric nematic phase for 3ECMe observed between untreated glass slides, (c) banded texture of the ferroelectric nematic phase, and (d) texture with parabolic domain walls for 5ECMe observed in cells with homeotropic anchoring.

The transitional properties of the *n*ECMeF series are reported in Table 3, with the data for 3ECMeF being extracted from our previous work.^31^ Only 1ECMeF exhibited the conventional nematic phase preceding the ferroelectric nematic phase, while the other compounds exhibited exclusively direct transitions between the isotropic and ferroelectric nematic phase. The N phase was again assigned by the observation of a characteristic schlieren texture with two- and four-brush point defects, Figure 4a, while the N_F_ phase was assigned on the basis of banded textures, a representative example of which is shown in Figure 4b.

**Table 3:** Transition Temperatures and Associated Entropy Changes for the *n*ECMeF Series^a^

compound	*T*_Cr–_/°C	*T*_N_F_N_/°C *T_N_F_I_/°C	*T*_NI_/°C	Δ*S*_Cr–_/*R*	*Δ*S*_N_F_N_/*R* Δ*S*_N_F_I_/*R*	Δ*S*_NI_/*R*	μ/D
1ECMeF	163	^b^117	184	12.0	^b^0.089	0.13	10.9
2ECMeF	159	*^b^136		13.1	*^b^1.21		11.8
3ECMeF	171	*^b^154		11.5	*^b^1.39		11.9
4ECMeF	139	*^b^109		13.1	*^b^1.31		12.2
5ECMeF	171	*^b^106		11.9	*^b^1.48		13.2
6ECMeF	136	*^b^116		11.8	*^b^1.63		11.6

a The calculated dipole moments, μ, are also listed.

b Values extracted from DSC cooling traces.

**Figure 4:**
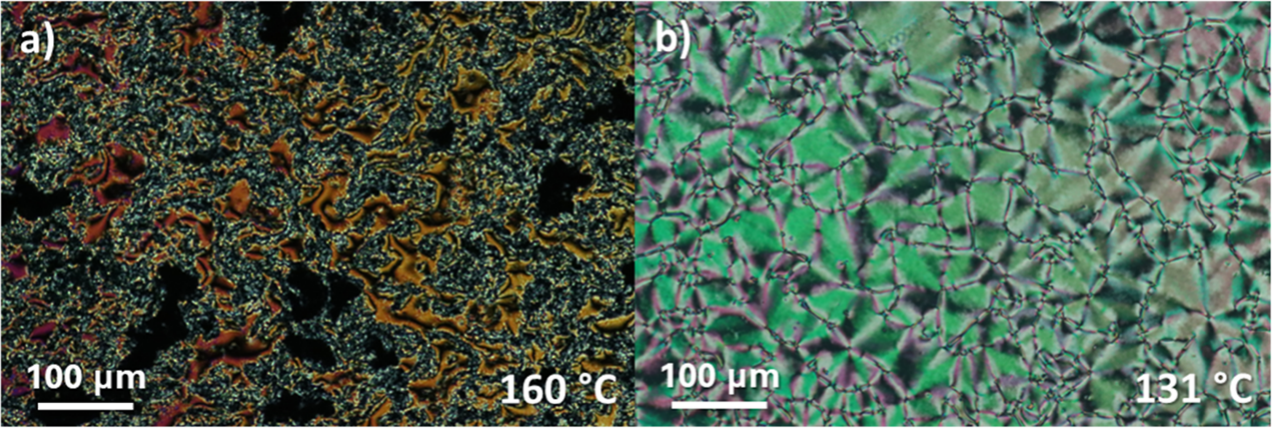
Polarized optical microscope textures for the *n*ECMeF series observed between untreated glass slides: (a) schlieren texture of the nematic phase for 1ECMeF and (b) banded texture of the ferroelectric nematic phase for 2ECMeF.

In order to confirm the N_F_ phase assignment, dielectric studies were carried out on a member of each series, 5ECMe and 4ECMeF specifically, to measure the temperature and frequency dependence of the real and imaginary components of the dielectric permittivity, as shown in Figure 5a,b. The direct transition between the isotropic liquid and ferroelectric nematic phase is marked in both samples by a sharp increase in the dielectric permittivity, ε, showing the emergence of a strong relaxation mode indicative of the polar nature of the phase. Specifically, the strong dielectric mode might be attributed to the collective movement of the polarization direction, phason mode.^64^ The measured values, as well as the abrupt emergence of the strong relaxation mode, are in excellent agreement with those reported for other ferroelectric nematogens.^24,25,30,36,37,41,43,48,64–66^ While there has been debate over the absolute value of the measured dielectric permittivity recently, the topic is complex and so beyond the scope of this present work.^17,64,67–72^ We can therefore by this analysis conclude with confidence that indeed our compound shows the N_F_ phase, while the absolute values of ε obtained should still be treated with some uncertainty, and the accuracy of the methodology continues to be investigated.

**Figure 5:**
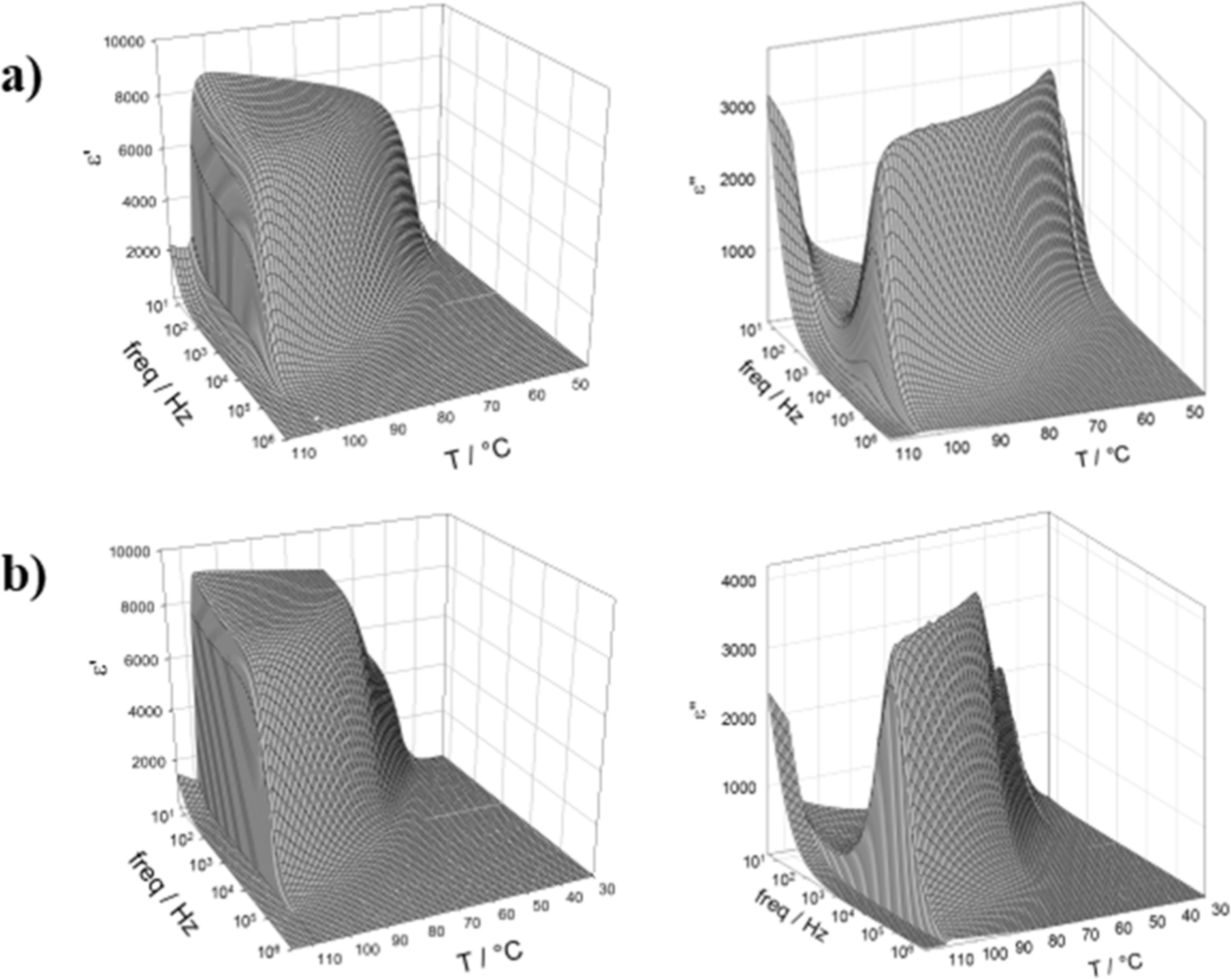
Real (left) and imaginary (right) parts of the complex dielectric permittivity measured as a function of temperature and frequency for (a) 5ECMe and (b) 4ECMeF in a 9.7 μm-thick cell with ITO electrodes and no alignment layer.

We now turn our attention to the effect of replacing the methoxy groups in the terminal ring of RM734 on the stability of both the conventional nematic phase and the ferroelectric nematic phase. The N_X_ phase recently reported in RM734 ^73,74^ has an extremely narrow temperature range, and is not the focus of this work, so is omitted in the discussions related to this material. The transition temperatures of the *n*ECMe series are compared to the literature compounds RM734 ^39^ and NT3.1 ^25^ in Figure 6a,b. The structure of NT3.1 is shown in Figure 7. Compound 1ECMe contains a methyl lateral group and has a value of *T*_NI_ of 218 °C and *T*_N_F_N_ of 71 °C—compared to RM734, there is stabilization of the N phase by 30 °C. The lateral methyl group of 1ECMe decreases the shape anisotropy less when compared to the methoxy lateral group of RM734, Figure 3, and this drives the increase in *T*_NI_. This effect is not replicated for *T*_N_F_N_ which falls by around 60 °C compared to RM734, presumably it is the lesser lateral bulk of the methyl group allowing antiparallel correlations to be favored to a greater extent which destabilizes the N_F_ phase. This decrease in the N_F_ phase could be considered surprising considering the predictions of a molecular model developed by Madhusudana to describe the ferroelectric nematic phase.^60^ A methoxy group is a better electron donor than a methyl group, and so it might be expected that RM734 would less favorably form the N_F_ phase; however, this is not the case. Modification of the molecular structure also sees a considerable change in the overall dipole of these compounds with RM734 having a longitudinal dipole moment of 11.4 D compared to a value of 9.91 D for 1ECMe. In addition, it is not only important to consider the electron-donating and electron-withdrawing effects of methoxy and methyl groups on the ring as a whole but also specifically on the position of the groups. This is highlighted when comparing 1ECMe to 2ECMe where the methoxy and methyl groups swap positions such that the methyl group is now *para* to the ester group of the terminal ring. This change sees the shape anisotropy decrease, by simultaneously increasing the molecular width while reducting the molecular length, and so *T*_NI_ is destabilized by 60 °C for 2ECMe. The *T*_N_F_N_ on the other hand is increased compared to 1ECMe by 50 °C, which suggests that the N_F_ phase is destabilized if the lateral bulk does not exceed a certain degree of biaxiality. It is clear that there is a fine balance between electronic and shape effects in driving the formation of the N_F_ phase, and these results suggest that for a more anisometric molecular shape, it is the electronic effects which have the greater influence. In this case when there is a methoxy terminus on the aromatic ring, there is a larger positive charge than when this is a methyl group, and this is unfavorable when considering the model described by Madhusudana.^60^ Furthermore, it has been shown in the literature that reducing the length of terminal alkyl/alkyloxy chains is considered favorable for the formation of the N_F_ phase.^39,44,75^ Despite stabilization compared to 1ECMe, the value of *T*_N_F_N_ for 2ECMe is still 5 °C lower than that of RM734. This observation suggests that the position of the two groups on the terminal ring of 2ECMe gives an overall less favorable electronic profile compared to RM734, hence the decrease in *T*_N_F_N_. The final modification in this set of materials is moving the methoxy lateral group from the terminal ring to the central aromatic ring while maintaining the methyl terminus, to give 3ECMe. This shift results in an increase of both *T*_NI_ and *T*_N_F_N_ compared to 2ECMe. The increase in *T*_NI_ of 15 °C suggests that the shape anisotropy of the molecule is enhanced despite having the same lateral group but in a different position. This is somewhat surprising considering our previous comparison of NT3.1 with RM734: the structural difference between the two is again a shift of the lateral methoxy group from the left-hand to the central aromatic ring, but now, almost identical values of *T*_NI_ are observed. This presumably reflects the effect that a terminal methyl group has on the ability of these compounds to pack efficiently. The stability of the N_F_ phase is enhanced in 3ECMe compared to 2ECMe, and this is in complete agreement with Madhusudana’s proposed model.^60^ Moving the lateral methoxy group reduces the electron donation effect into the terminal aromatic ring and, instead, increases this effect into the central aromatic ring. This effect combinatorially reduces the amplitude of the charge density wave at the terminal of the molecule while increasing that of the interior wave, and this stabilizes the N_F_ phase by promoting the parallel correlation of the calamitic molecules. This observation is in good agreement with our report on moving an alkyloxy chain from the terminal ring to the middle ring.^25^ The clearing temperature of 3ECMe is 171 °C, which is lower than that of NT3.1,^25^ at 189 °C, and this can be attributed to the decrease in shape anisotropy due to the smaller size of the terminal methyl group compared to a methoxy. Interestingly, for 3ECMe, there is now an increase in *T*_N_F_N_ of 27 °C compared to that of NT3.1. This was not the case when comparing 2ECMe and RM734, despite the alteration to their respective termini being the same. Presumably having the methoxy in the middle ring when the terminal ring solely contains a methyl group strongly drives favorable interactions between the molecules such that they align in a parallel manner or destabilizes the interactions driving antiparallel packing. Thus, the favorable shape and electronic profile in this case lead to a stabilization of the N_F_ phase, unlike the case of 2ECMe vs RM734. Evidentially, these materials can be very sensitive to the position of the lateral and terminal substituents in terms of their packing arrangement, such that even a small structural change can have a large effect on the transition temperatures.

**Figure 6:**
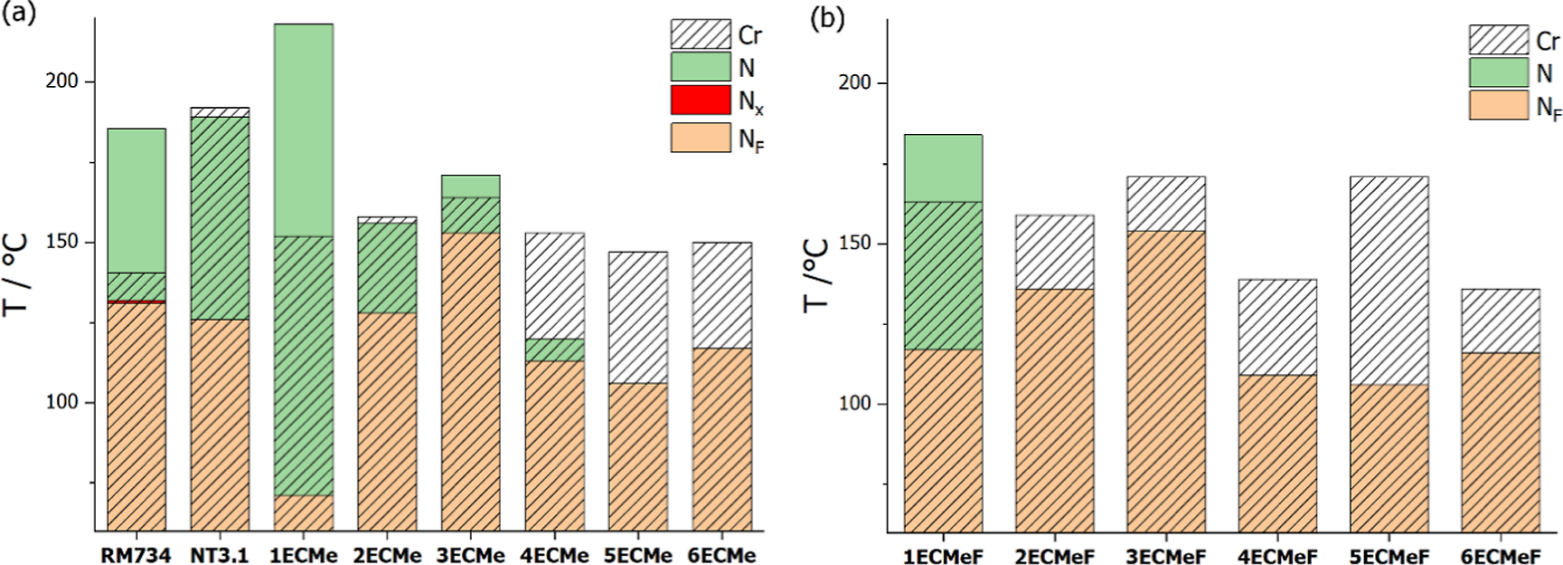
Transition temperatures of the (a) *n*ECMe series compared to RM734 and NT3.1 ^30,39^ and (b) *n*ECMeF series. The dashed areas show the stability range of crystal phases.

**Figure 7:**
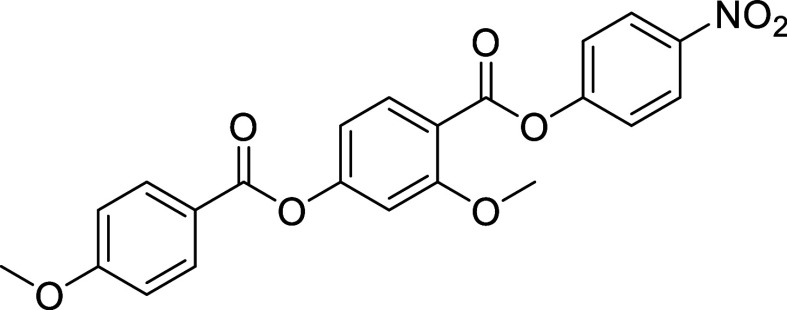
Molecular structure of compound NT3.1.^25^

4ECMe and 5ECMe can be directly compared to 1ECMe and 2ECMe, respectively, to assess the effect of an additional methoxy group in the central aromatic ring. This addition sees *T*_NI_ greatly reduced such that 4ECMe exhibits a conventional nematic phase, albeit around 100 °C lower than 1ECMe, but 5ECMe shows a direct N_F_–I transition, with the N phase being extinguished due to the decrease in shape anisotropy. The additional methoxy group has a weaker effect on the ferroelectric nematic phase in terms of decreasing the transition temperatures, and in the case of 4ECMe, the stability of the N_F_ phase actually increases with the additional lateral bulk. This suggests that the ability of these materials to pack in a parallel fashion is related to both electronic effects and these changes in shape. The *T*_N_F_N_ of 4ECMe is 113 °C, which is around 40 °C higher than that of 1ECMe, whereas 5ECMe shows a 30 °C decrease compared to 2ECMe. The addition of a methoxy group to the central ring increases the charge density of this fragment which according to Madhusudana’s model sees an increase in the stability of the N_F_ phase.^60^ This will, of course, be true for both pairs of molecules, but 1ECMe has the most anisometric structure, and the overall biaxiality is less, to the extent that the N_F_ phase is destabilized. This combination of the electronic and shape considerations gives rise to the overall increase in the stability of the N_F_ phase for 4ECMe. 2ECMe, however, is more biaxial, and so the addition of more lateral bulk in 5ECMe sees shape effects dominate and so the N_F_ phase is destabilized. The final compound in this series, 6ECMe, has two methyl groups in the terminal aromatic ring, and *T*_N_F_N_ is stabilized in this material compared to both 4ECMe and 5ECMe which have a combination of one methoxy group and one methyl group. This is in agreement with Madhusudana’s model with the magnitude of the charge density wave being reduced due to the less electron-donating nature of the methyl group compared to the methoxy group.^60^ The comparable compound to 6ECMe without the middle methoxy group, named 4 ^32^ and shown in Figure 8, was reported not to exhibit the ferroelectric nematic phase. This presumably is due to the methyl lateral group not providing sufficient lateral bulk to allow for favorable parallel packing of the molecules. However, compound 4 did exhibit a conventional nematic phase at 182 °C, which is a stabilization of at least 65 °C compared to 6ECMe and is in excellent agreement with the observations for 1ECMe-4ECMe and 2ECMe-5ECMe comparisons.

**Figure 8:**
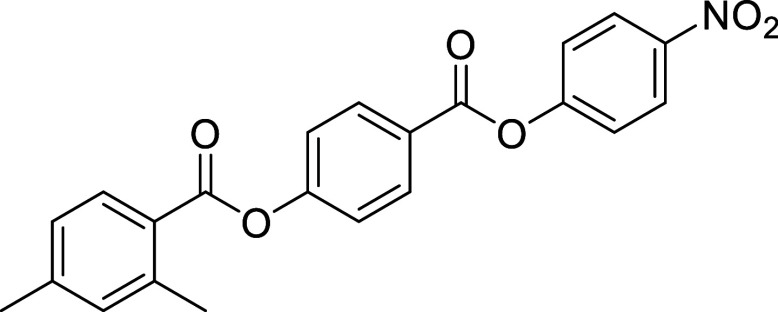
Molecular structure of compound 4.^32^

In Figure 6, the transition temperatures of the *n*ECMe and *n*ECMeF series are also compared to show the effect of adding a fluorine atom *ortho* to the terminal nitro group. Of the *n*ECMeF series, only 1ECMeF exhibited the conventional nematic phase, and this had a value of *T*_NI_ of 184 °C, 34 °C less than 1ECMe. This decrease is in excellent agreement with a number of other RM734-type compounds that we have reported on previously.^25,35,41,42^ The effect of the fluorination is two-fold: the larger steric bulk associated with the fluorine causes a decrease in the shape anisotropy of these compounds, and in addition, there is a change in the electronic distribution with the negative charge at the terminal of the molecule more widely spread. The ferroelectric nematic phase on the other hand is significantly stabilized in the case of 1ECMeF, with *T*_N_F_N_ increasing by almost 50 °C. This supports our earlier observation that with regard to N_F_ phase stabilization, electronic effects tend to dominate over shape contributions when the material is more anisometric. 2ECMeF and 3ECMeF both also saw a stabilization of the N_F_ phase with the addition of the fluorine *ortho* to the NO_2_ group compared to the corresponding *n*ECMe compounds, albeit these increases were much more modest: 8 and 1 °C, respectively, and both of these compounds exhibited direct N_F_–I transitions. The increase in *T*_N_F_N/I_ on addition of a fluorine atom *ortho* to the NO_2_ group in this type of molecule has been interpreted previously within the framework of the model proposed by Madhusudana.^60^ The addition of the lateral fluorine substituent spreads electron density more evenly around the terminal, and this reduces the amplitude of the charge density wave which accounts for the observed stabilization of the N_F_ phase. The other three members of the *n*ECMeF series (*n* = 4, 5, and 6) possess a methoxy group in the central aromatic ring as well as a lateral substituent in the left-hand terminus. For these compounds, the addition of a fluorine atom no longer causes a stabilization of the N_F_ phase when compared to the corresponding members of the *n*ECMe series; instead, there tends to be a destabilization. The largest decrease is between 4ECMeF and 4ECMe, for which *T*_N_F_I_ is 109 and 113 °C, respectively, while 5ECMeF and 6ECMeF showed values of *T*_N_F_I_ essentially equal to those of the corresponding members of the *n*ECMe series. It is therefore apparent that there is no longer a consistent effect on the stability of the N_F_ phase upon the addition of this F atom. This is in agreement with our previous observations on materials with multiple lateral groups^30,35^ and must be related to a detrimental decrease in shape anisotropy offsetting the positive electronic effects endowed by the F, namely, an increase in longitudinal dipole and the aforementioned spreading of electronic charge across the ring lowering the amplitude of the charge density wave. It seems that having multiple lateral groups causes these molecules to no longer have optimal biaxiality for packing into parallel structures. This also highlights the importance of intermolecular interactions between mesogens in the formation of the N_F_ phase as in this case they are disrupted by additional lateral groups. These data suggest that when there is only a single lateral group, then adding a fluorine tends to have a stabilizing effect on the N_F_ phase, whereas when there are multiple lateral groups, then there is a much more variable effect on the N_F_ phase, and this depends on both the electronic distribution across the molecules as well as the shape. Gibb *et al*.^57^ through systematic fluorination along the molecular backbone also found that the lateral intermolecular interactions between the mesogens, such as offset π–π stacking of the biphenyl units, were critical to the observation of the N_F_ phase. They also highlighted that while the simple models for the N_F_ phase give insights as to the rationale behind the observed phase transitions, there are some limitations which need further investigation. This further highlights the need for more research into this class of compounds in order to better understand, at a fundamental level, what is driving this variety in phase stability.

## Conclusions

Both newly synthesized series, *n*ECMe and *n*ECMeF, exhibited monotropic transitions to the ferroelectric nematic phase regardless of the position of the methyl group. The effect of exchanging the methoxy groups in the conventional RM734 structure for methyl groups varies depending on the position of this change and the other structural features of the molecule. Broadly, when the methyl group is in a lateral position, *T*_NI_ increases compared to the methoxy equivalent due to a reduction in the molecular breadth, whereas when the methyl group is in a terminal position, *T*_NI_ decreases since the molecular length is instead reduced. The effect on *T*_N_F_N/I_, however, had a less distinct trend with the values being very sensitive to the overall molecular structure. This is particularly apparent when comparing 2ECMe and 3ECMe which both have a terminal methyl group and a lateral methoxy group; the *T*_N_F_N_ of 3ECMe is 25 °C higher than that of 2ECMe. Now considering the addition of fluorine *ortho* to the terminal nitro group, there is a consistent decrease in *T*_NI_ due to the increase in steric lateral bulk, which reduces the shape anisotropy of the molecules. In terms of *T*_N_F_N/I_, when there is only a single lateral substituent, the value of *T*_N_F_N/I_ increases with the addition of fluorine, but when there are multiple lateral substituents, the N_F_ transition temperatures instead stay the same or decrease. This suggests that the discussion that larger dipole moments will drive an increase in the stability of the N_F_ phase is rather simplistic in nature. Indeed, the properties of these materials can be justified, in general, by the model of Madhusudana^60^ and are sensitive to the electronic distribution within the compounds.

## Supplementary Material



## Notes

Synthetic routes used to prepare the *n*ECMe and *n*ECMeF series, including detailed descriptions of the preparation of these compounds and the structural characterization data for all intermediates and final products (PDF)

## §

School of Pharmacy, Applied Sciences and Public Health, Robert Gordon University, Aberdeen AB10 7GJ, U.K

## ∥

Deceased 14th January 2025.
