# Supplementary material for: The Influence of Methyl Groups on the Formation of the Ferroelectric Nematic Phase
Source: ACS Omega. 2025 May 27;10(22):23609–19. doi: 10.1021/acsomega.5c02500 (PMC12163782; doi:10.1021/acsomega.5c02500)
Supplement: Supplementary file 1 [file ao5c02500_si_001.pdf]

The influence of methyl groups on the formation of the ferroelectric nematic phase

Ewan Cruickshank[1,‡,*], Rebecca Walker[1], Magdalena M. Majewska[2], Ewa Gorecka[2], Damian Pociecha[2], John M.D. Storey[1] & Corrie T. Imrie[1,†]

[1]Department of Chemistry, University of Aberdeen, Old Aberdeen, AB24 3UE, U.K.

[2]Faculty of Chemistry, University of Warsaw, Zwirki i Wigury 101, 02-089 Warsaw, Poland

‡Present Address: School of Pharmacy and Life Sciences, Robert Gordon University, Aberdeen, AB10 7GJ, U.K.

†Deceased 14th January 2025

*Author for correspondence: e.cruickshank2@rgu.ac.uk

Experimental Procedures

Synthesis

Reagents

All reagents and solvents that were available commercially were purchased from Sigma Aldrich, Fisher Scientific or Fluorochem and were used without further purification unless otherwise stated.

Thin Layer Chromatography

Reactions were monitored using thin layer chromatography, and the appropriate solvent system, using aluminium-backed plates with a coating of Merck Kieselgel 60 F254 silica which were purchased from Merck KGaA. The spots on the plate were visualised by UV light (254 nm) or by oxidation using either a potassium permanganate stain or iodine dip.

Column Chromatography

For normal phase column chromatography, the separations were carried out using silica gel grade 60 Å, 40-63 µm particle size, purchased from Fluorochem and using an appropriate solvent system.

Structure Characterisation

All final products and intermediates that were synthesised were characterised using ^{1}H NMR, ^{13}C NMR, infrared spectroscopies and mass spectrometry. The NMR spectra were recorded on a 400 MHz Bruker Avance III HD NMR spectrometer. The infrared spectra were recorded on a Perkin Elmer Spectrum Two FTIR spectrometer with an ATR diamond cell. High-resolution mass spectrometry was carried out using a Waters XEVO G2 QTof mass spectrometer by Dr. Jayne McCaskill at the University of Aberdeen.

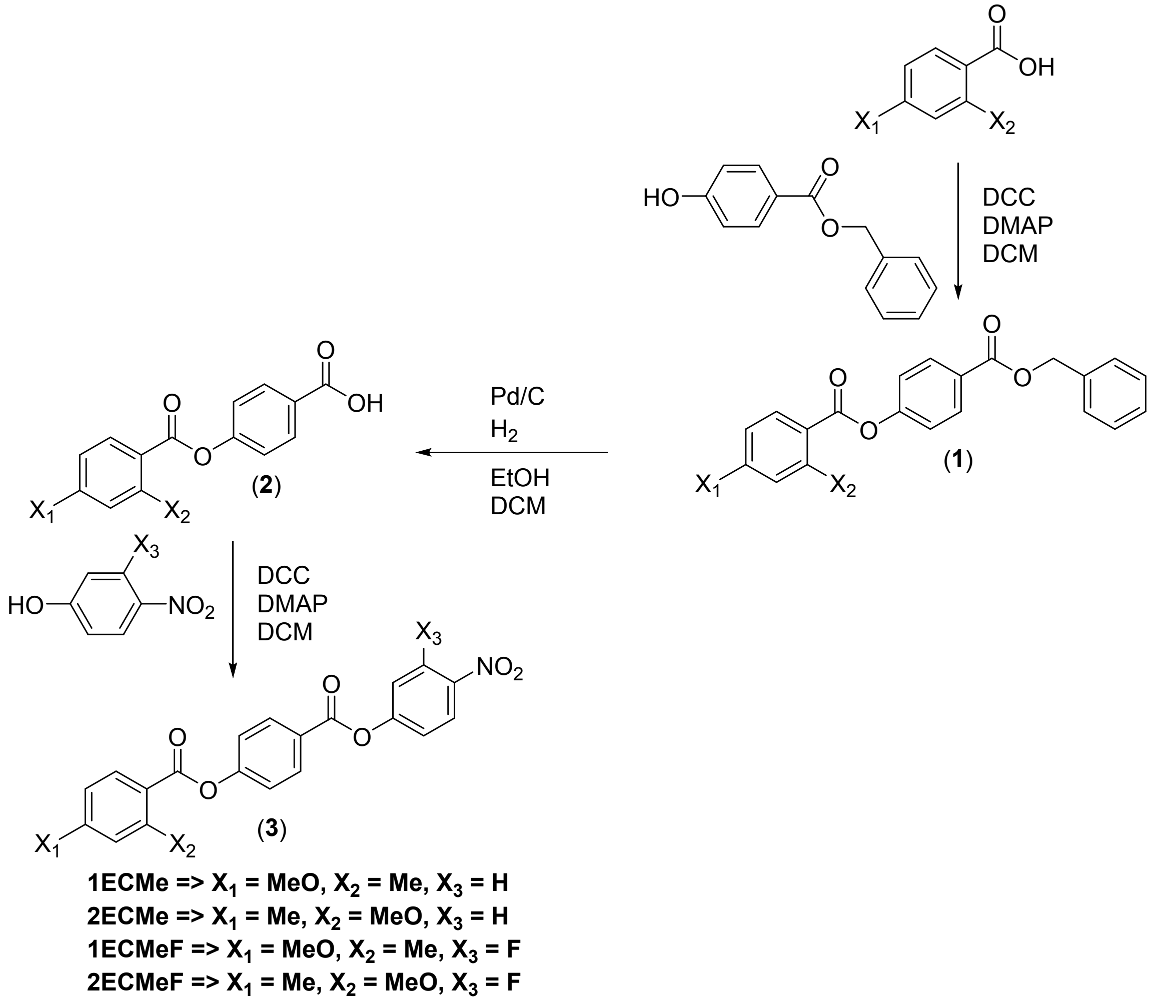

Scheme 1. Synthesis of 1ECMe, 2ECMe, 1ECMeF and 2ECMeF.

Compound 1

To a pre-dried flask flushed with argon, 4-methoxy-2-methylbenzoic acid (1 eq) or 2-methoxy-4-methylbenzoic acid (1 eq), benzyl 4-hydroxybenzoate (1.1 eq) and 4-dimethylaminopyridine (0.13 eq) were added. The solids were solubilised with dichloromethane (80 mL) and stirred for 10 min before *N,N′*-dicyclohexylcarbodiimide (1.3 eq) was added to the flask and the reaction was allowed to proceed overnight. The quantities of the reagents used in each reaction are listed in **Table S1**. The extent of the reaction was monitored by TLC using an appropriate solvent system (RF values quoted in the product data). The precipitate which formed was removed by vacuum filtration and the filtrate collected. The collected solvent was evaporated under vacuum to leave a white solid which was recrystallised from hot ethanol (100 mL).

Table S1. Quantities of reagents used in the syntheses of **Compound 1**

Product	4-Methoxy-2-methylbenzoic acid /*2-Methoxy-4-methylbenzoic acid	Benzyl 4-hydroxybenzoate	4-Dimethylaminopyridine	*N,N′*-Dicyclohexylcarbodiimide
1.1	3.00 g, 0.0181 mol	4.54 g, 0.0199 mol	0.332 g, 2.35×10^{-3} mol	5.61 g, 0.0235 mol

1.2	*3.00 g, 0.0181 mol	4.54 g, 0.0199 mol	0.332 g, 2.35×10^{-3} mol	5.61 g, 0.0235 mol

1.1 4-[(Benzyloxy)carbonyl]phenyl 4-methoxy-2-methylbenzoate

Yield: 5.82 g, 85.4 %. RF: 0.56 (40 % ethyl acetate: 60 % 40:60 petroleum ether). M.P = 109 °C

v_{max}/cm^{-1}: 2927, 1735, 1705, 1613, 1568, 1498, 1454, 1412, 1377, 1313, 1300, 1281, 1252, 1210, 1165, 1137, 1123, 1098, 1056, 1027, 1015, 1002, 982, 926, 874, 834, 819, 764, 732, 689, 656, 634, 623, 582, 561, 521, 507, 461, 451

δ_H/ppm (400 MHz, DMSO-d_6): 8.10 (3 H, m, Ar-H), 7.49 (2 H, d, J 7.0 Hz, Ar-H), 7.41 (5 H, m, Ar-H), 6.96 (2 H, m, Ar-H), 5.37 (2 H, s, O-$\underline{CH_2}$-Ar), 3.85 (3 H, s, O-$\underline{CH_3}$), 2.58 (3 H, s, Ar-CH_3)

δ_C/ppm (100 MHz, DMSO-d_6): 165.41, 164.53, 163.34, 155.10, 144.10, 136.55, 133.99, 131.32, 129.00, 128.60, 128.44, 127.46, 123.09, 120.01, 117.61, 112.18, 66.75, 55.98, 22.31

1.2 4-[(Benzyloxy)carbonyl]phenyl 2-methoxy-4-methylbenzoate

Yield: 4.94 g, 72.4 %. RF: 0.580 (40 % ethyl acetate: 60 % 40:60 petroleum ether). M.P = 59 °C

v_{max}/cm^{-1}: 2951, 1740, 1711, 1604, 1586, 1573, 1497, 1466, 1455, 1446, 1418, 1370, 1281, 1266, 1231, 1194, 1166, 1108, 1096, 1081, 1023, 1007, 938, 918, 870, 859, 834, 824, 792, 771, 764, 750, 722, 701, 692, 632, 606, 560, 518, 507, 453

δ_H/ppm (400 MHz, DMSO-d_6): 8.08 (2 H, d, 8.7 Hz, Ar-H), 7.85 (1 H, d, J 8.0 Hz, Ar-H), 7.49 (2 H, d, J 7.2 Hz, Ar-H), 7.40 (5 H, m, Ar-H), 7.07 (1 H, d, Ar-H), 6.96 (1 H, dd, J 7.0 Hz, Ar-H), 5.37 (2 H, s, O-$\underline{CH_2}$-Ar), 3.86 (3 H, s, O-$\underline{CH_3}$), 2.40 (3 H, s, Ar-CH_3)

δ_C/ppm (100 MHz, DMSO-d_6): 165.40, 163.54, 159.93, 155.05, 146.35, 136.54, 132.29, 131.36, 129.01, 128.61, 128.46, 127.49, 123.00, 121.43, 115.41, 113.85, 66.76, 56.34, 22.02

Compound 2

To a pre-dried flask flushed with argon, **Compound 1** (1 eq) was dissolved in a mixture of dichloromethane and ethanol and stirred. The mixture was sparged with argon and 5 % Pd/C catalyst was added. The argon atmosphere was evacuated under vacuum and replaced by hydrogen gas. The quantities of the reagents used in each reaction are listed in **Table S2**. The reaction was allowed to proceed for 4 h at room temperature, with the extent of the reaction monitored by TLC using an appropriate solvent system (RF values quoted in the product data). After the reaction was completed, the hydrogen gas was evacuated under vacuum and the flask purged using argon. The mixture was filtered through Celite using copious amounts of dichloromethane, and the collected solvent was evaporated under vacuum to leave a white solid which was carried forwards without any further purification.

Table S2. Quantities of reagents used in the syntheses of **Compound 2**

Product	Compound 1.1/1.2*	5 % Palladium on Carbon	Dichloromethane	Ethanol
2.1	5.50 g, 0.0146 mol	0.311 g, 2.92×10^{-3} mol	90 mL	90 mL

2.2	*4.70 g, 0.0125 mol	0.266 g, 2.50×10^{-3} mol	70 mL	70 mL

2.1 4-(4-Methoxy-2-methylbenzoyloxy)benzoic acid

Yield: 1.12 g, 26.8 %. RF: 0.06 (40 % ethyl acetate: 60 % 40:60 petroleum ether).

T_{CrN} 225 °C T_{NI} (210 °C)

v_{max}/cm^{-1}: 2931, 1734, 1679, 1604, 1563, 1507, 1447, 1428, 1377, 1319, 1296, 1246, 1199, 1165, 1124, 1055, 1024, 925, 879, 819, 763, 727, 689, 650, 634, 617, 547, 523, 507, 454, 407

δ_H/ppm (400 MHz, DMSO-d_6): 13.04 (1 H, br, (C=O)-OH), 8.11 (2 H, d, J 8.6 Hz, Ar-H), 8.03 (2 H, d, J 8.7 Hz, Ar-H), 7.38 (2 H, d, J 8.7 Hz, Ar-H), 6.96 (2 H, m, Ar-H), 3.85 (3 H, s, O-$\underline{CH_3}$), 2.96 (3 H, s, Ar-$\underline{CH_3}$)

δ_C/ppm (100 MHz, DMSO-d_6): 167.16, 164.63, 163.30, 154.65, 144.03, 133.95, 131.30, 128.78, 122.78, 120.12, 117.61, 112.18, 55.98, 22.31

2.2 4-(2-Methoxy-4-methylbenzoyloxy)benzoic acid

Yield: 3.02 g, 84.4 %. RF: 0.05 (40 % ethyl acetate: 60 % 40:60 petroleum ether). M.P = 202 °C

v_{max}/cm^{-1}: 2850, 1746, 1679, 1601, 1573, 1501, 1470, 1422, 1315, 1289, 1267, 1238, 1192, 1163, 1141, 1101, 1029, 1006, 955, 942, 883, 847, 826, 812, 779, 762, 721, 692, 656, 605, 548, 529, 504, 450, 414

δ_H/ppm (400 MHz, DMSO-d_6): 13.04 (1 H, br, (C=O)-OH), 8.03 (2 H, d, J 8.7 Hz, Ar-H), 7.85 (1 H, d, J 7.9 Hz, Ar-H), 7.36 (2 H, d, J 8.7 Hz, Ar-H), 7.08 (1 H, s, Ar-H), 6.92 (2 H, d, J 7.9 Hz, Ar-H), 3.87 (3 H, s, O-$\underline{CH_3}$), 2.40 (3 H, s, Ar-$\underline{CH_3}$)

δ_C/ppm (100 MHz, DMSO-d_6): 167.12, 163.66, 159.88, 154.62, 146.26, 132.24, 131.35, 128.74, 122.69, 121.43, 115.54, 113.85, 56.34, 22.02

Compound 3

To a pre-dried flask flushed with argon, **Compound 2** (1 eq), 4-nitrophenol (1.2 eq for 3.1 and 1.5 eq for 3.3) or 3-fluoro-4-nitrophenol (1.2 eq for 3.2 and 1.5 eq for 3.4), and *N*-(3-Dimethylaminopropyl)-*N'*-ethylcarbodiimide hydrochloride (1.5 eq) were added to the flask. The solids were solubilised with dichloromethane (30 mL) and stirred for 30 min before 4-dimethylaminopyridine (0.15 eq) was added. The quantities of the reagents used in each reaction are listed in **Table S3**. The temperature of the reaction mixture was increased to room temperature and the reaction was allowed to proceed overnight. The white precipitate which formed was removed by vacuum filtration and the filtrate collected. The solvent was removed under vacuum and the crude product was purified using a silica gel column with an appropriate solvent system (RF values quoted in product data). The eluent fractions of interest were evaporated under vacuum to leave a white solid which was recrystallised from hot ethanol (100 mL).

Table S3. Quantities of reagents used in the syntheses of **Compound 3**

Product	Compound 2.1/2.2*	4-Nitrophenol/*3-Fluoro-4-nitrophenol	*N*-(3-Dimethylaminopropyl)-*N′*-ethylcarbodiimide hydrochloride	4-Dimethylaminopyridine
3.1	0.300 g, 1.05×10^{-3} mol	0.175 g, 1.26×10^{-3} mol	0.303 g, 1.58×10^{-3} mol	0.019 g, 1.58×10^{-4} mol
3.2	0.300 g, 1.05×10^{-3} mol	*0.198 g, 1.26×10^{-3} mol	0.303 g, 1.58×10^{-3} mol	0.019 g, 1.58×10^{-4} mol
3.3	*0.300 g, 1.05×10^{-3}	0.220 g, 1.58×10^{-3} mol	0.303 g, 1.58×10^{-3} mol	0.019 g, 1.58×10^{-4} mol
3.4	*0.300 g, 1.05×10^{-3}	*0.248 g, 1.58×10^{-3} mol	0.303 g, 1.58×10^{-3} mol	0.019 g, 1.58×10^{-4} mol

3.1 4-[(4-Nitrophenoxy)carbonyl]phenyl 4-methoxy-2-methylbenzoate

Yield: 0.104 g, 24.2 %. RF: 0.32 (100 % dichloromethane).

T_{CrN} 152 °C T_{N_FN} (71 °C) T_{NI} 218 °C

ν_{max}/cm^{-1}: 2977, 1733, 1607, 1573, 1525, 1514, 1470, 1413, 1357, 1332, 1266, 1242, 1203, 1163, 1127, 1108, 1071, 1058, 1040, 1026, 1008, 939, 864, 853, 814, 765, 752, 743, 719, 682, 671, 630, 599, 568, 509, 487, 450, 405

δ_H/ppm (400 MHz, DMSO-d_6): 8.37 (2 H, d, 8.9 Hz, Ar-H), 8.25 (2 H, d, 8.7 Hz, Ar-H), 8.15 (1 H, d, J 8.5 Hz, Ar-H), 7.66 (2 H, d, 8.9 Hz, Ar-H), 7.53 (2 H, d, 8.7 Hz, Ar-H), 6.99 (3 H, m, Ar-H), 3.86 (3 H, s, O-$\underline{CH_3}$), 2.60 (3 H, s, Ar-$\underline{CH_3}$)

δ_C/ppm (100 MHz, DMSO-d_6): 164.47, 163.77, 163.39, 155.96, 155.84, 145.67, 144.18, 134.06, 132.20, 126.20, 125.79, 123.86, 123.36, 119.95, 117.64, 112.22, 56.01, 22.32

MS = $[2M+Na]^+$: Calculated for $C_{44}H_{34}N_2O_{14}Na$: 837.1908. Found: 837.1877. Difference: 3.7 ppm

3.2 4-[(3-Fluoro-4-nitrophenoxy)carbonyl]phenyl 4-methoxy-2-methylbenzoate

Yield: 0.135 g, 30.2 %. RF: 0.34 (100 % dichloromethane).

T_{CrN} 163 °C T_{N_FN} (117 °C) T_{NI} 184 °C

ν_{max}/cm^{-1}: 2973, 1732, 1606, 1574,1525, 1512, 1469, 1413, 1357, 1332, 1241, 1202, 1162, 1126, 1109, 1053, 1024, 1011, 939, 863, 853, 814, 765, 752, 719, 683, 671, 630, 599, 568, 509, 487

δ_H/ppm (400 MHz, DMSO-d_6): 8.32 (1 H, dd, J 8.8 Hz, 8.7 Hz, Ar-H), 8.24 (2 H, d, J 8.7 Hz, Ar-H), 8.15 (1 H, d, 8.6 Hz, Ar-H), 7.79 (1 H, dd, 12.0 Hz, 2.4 Hz, Ar-H), 7.54 (2 H, d, 8.7 Hz, Ar-H), 7.50 (1 H, m, Ar-H), 6.98 (2 H, m, Ar-H), 3.91 (3 H, s, O-$\underline{CH_3}$), 2.60 (3 H, s, Ar-$\underline{CH_3}$)

δ_F/ppm (376 MHz, DMSO-d_6): -115.37 (1 F, s, Ar-F)

δ_C/ppm (100 MHz, DMSO-d$_6$): 164.45, 163.44, 163.40, 156.03 (d, J 11.2 Hz), 155.94, 155.68 (d, J 262.8 Hz), 144.19, 135.21 (d, J 7.3 Hz), 134.06, 132.26, 128.00 (d, J 2.0 Hz), 125.94, 123.39, 119.93, 119.71 (d, J 3.8 Hz), 117.64, 113.32 (d, J 23.8 Hz), 112.23, 56.01, 22.32

MS = $[2M+Na]^+$: Calculated for $C_{44}H_{32}N_2O_{14}F_2Na$: 873.1719. Found: 873.1683. Difference: 4.1 ppm

3.3 4-[(4-Nitrophenoxy)carbonyl]phenyl 2-methoxy-4-methylbenzoate

Yield: 0.113 g, 26.4 %. RF: 0.15 (100 % dichloromethane).

T_{CrI} 158 °C T_{N_FN} (128 °C) T_{NI} (156 °C)

v_{max}/cm^{-1}: 1745, 1613, 1590, 1518, 1496, 1466, 1407, 1340, 1290, 1257, 1237, 1197, 1176, 1156, 1131, 1111, 1046, 1028, 1008, 963, 886, 862, 855, 822, 768, 754, 743, 721, 689, 666, 639, 626, 589, 544, 529, 505, 480 451

δ_H/ppm (400 MHz, $CDCl_3$): 8.36 (2 H, d, 9.0 Hz, Ar-H), 8.24 (2 H, d, 8.5 Hz, Ar-H), 7.88 (1 H, d, J 7.9 Hz, Ar-H), 7.65 (2 H, d, J 9.0 Hz, Ar-H), 7.50 (2 H, d, J 8.5 Hz, Ar-H), 7.09 (1 H, s, Ar-H), 6.94 (1 H, d, J 8.0 Hz, Ar-H), 3.89 (3 H, s, O-$\underline{CH_3}$), 2.41 (3 H, s, Ar-$\underline{CH_3}$)

δ_C/ppm (100 MHz, $CDCl_3$): 163.75, 163.44, 160.00, 155.96, 155.77, 146.47, 145.67, 132.35, 132.24, 126.24, 125.78, 123.86, 123.26, 121.45, 115.30, 113.87, 56.37, 22.04

MS = $[M+Na]^+$: Calculated for $C_{22}H_{17}NO_7Na$: 430.0903. Found: 430.0901. Difference: 0.5 ppm

3.4 4-[(3-Fluoro-4-nitrophenoxy)carbonyl]phenyl 2-methoxy-4-methylbenzoate

Yield: 0.138 g, 30.9 %. RF: 0.14 (100 % dichloromethane).

T_{CrI} 159 °C T_{N_FI} (136 °C)

v_{max}/cm^{-1}: 1739, 1611, 1602, 1525, 1499, 1413, 1378, 1352, 1290, 1251, 1206, 1156, 1145, 1094, 1057, 1040, 1022, 965, 935, 882, 847, 815, 762, 749, 722, 690, 669, 628, 595, 534, 501, 455

δ_H/ppm (400 MHz, $CDCl_3$): 8.26 (2 H, d, 8.8 Hz, Ar-H), 8.20 (1 H, dd, J 8.8 Hz, 8.6 Hz, Ar-H), 7.44 (2 H, d, 8.8 Hz, Ar-H), 7.30 (1 H, dd, 11.2 Hz, 2.4 Hz, Ar-H), 7.23 (1 H, ddd, J 8.8 Hz, 2.4 Hz, 1.33 Hz, Ar-H), 6.57 (2 H, d, 10.37 Hz, Ar-H), 3.89 (3 H, s, O-$\underline{CH_3}$)

δ_F/ppm (376 MHz, $CDCl_3$): -115.39 (1 F, s, Ar-F)

δ_C/ppm (100 MHz, $CDCl_3$): 163.42, 160.01, 156.04 (d, J 11.1 Hz), 155.87, 155.68 (d, J 262.7 Hz), 146.48, 135.21 (d, J 7.2 Hz), 132.35, 132.30, 127.99 (d, J 2.0 Hz), 125.98, 123.29, 121.45, 119.70 (d, J 3.8 Hz), 115.27, 113.86, 113.31 (d, J 23.8 Hz), 56.49, 56.35, 22.03, 19.01

MS = $[M+Na]^+$: Calculated for $C_{22}H_{16}NO_7FNa$: 448.0808. Found: 448.0801. Difference: 1.6 ppm

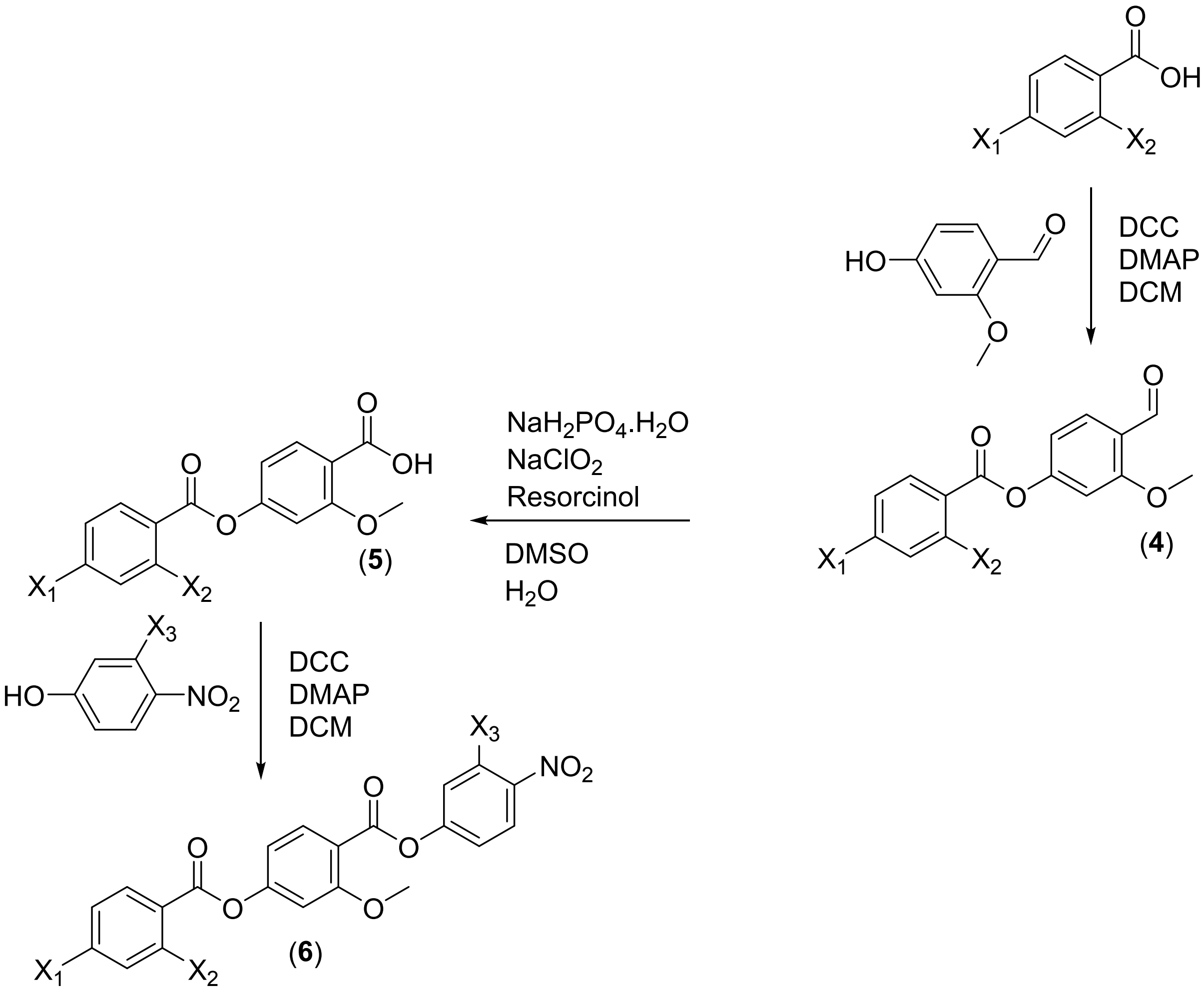

3ECMe => X_1 = Me, X_2 = H, X_3 = H
4ECMe => X_1 = MeO, X_2 = Me, X_3 = H
5ECMe => X_1 = Me, X_2 = MeO, X_3 = H
6ECMe => X_1 = Me, X_2 = Me, X_3 = H
3ECMeF => X_1 = Me, X_2 = H, X_3 = F
4ECMeF => X_1 = MeO, X_2 = Me, X_3 = F
5ECMeF => X_1 = Me, X_2 = MeO, X_3 = F
6ECMeF => X_1 = Me, X_2 = Me, X_3 = F

Scheme 2. Synthesis of 3ECMe-6ECMe and 3ECMeF-6ECMeF.

Compound 4

To a pre-dried flask flushed with argon, the required benzoic acid (1 eq), 4-hydroxy-2-methoxybenzaldehyde (1.1 eq) and 4-dimethylaminopyridine (0.13 eq or 0.15 eq for **Compounds 4.2** and **4.3**) were added. The solids were solubilised with dichloromethane (100 mL) and tetrahydrofuran (50 mL) while being stirred for 10 min before *N*,*N'*-dicyclohexylcarbodiimide (1.3 eq, 1.4 eq for **Compound 4.1** or 1.5 eq for **Compounds 4.2 or 4.3**) was added to the flask and the reaction was allowed to proceed overnight. The quantities of the reagents used in each reaction are listed in **Table S4**. The extent of the reaction was monitored by TLC using an appropriate solvent system (RF values quoted in the product data). The precipitate which formed was removed by vacuum filtration and the filtrate collected. The collected solvent was evaporated under vacuum to leave a solid which was recrystallised from hot ethanol (200 mL).

Table S4. Quantities of reagents used in the syntheses of **Compound 4**

Product	Benzoic acid Name	Benzoic Acid	4-Hydroxy-2-methoxybenzaldehyde	4-Dimethylaminopyridine	*N,N'*-Dicyclohexylcarbodiimide
4.1	4-Methylbenzoic acid	2.50 g, 0.0184 mol	3.07 g, 0.0202 mol	0.292 g, 2.39×10^{-3} mol	4.93 g, 0.0256 mol
4.2	4-Methoxy-2-methylbenzoic acid	3.00 g, 0.0181 mol	3.03 g, 0.0199 mol	0.332 g, 2.72×10^{-3} mol	5.61 g, 0.0272 mol
4.3	2-Methoxy-4-methylbenzoic acid	3.00 g, 0.0181 mol	3.03 g, 0.0199 mol	0.332 g, 2.72×10^{-3} mol	5.61 g, 0.0272 mol
4.4	2,4-Dimethylbenzoic acid	3.00 g, 0.0200 mol	3.35 g, 0.0220 mol	0.318 g, 2.60×10^{-3} mol	5.36 g, 0.0260 mol

4.1 4-Formyl-3-methoxyphenyl 4-methylbenzoate

White Solid. Yield: 3.35 g, 67.4 %. RF: 0.55 (40 % ethyl acetate:60 % 40:60 petroleum ether). M.P =117 °C

ν_{max}/cm^{-1}: 2869, 1739, 1679, 1599, 1587, 1493, 1474, 1465, 1417, 1403, 1379, 1262, 1248, 1200, 1182, 1157, 1120, 1099, 1063, 1027, 1017, 945, 872, 838, 822, 806, 788, 743, 686, 668, 627, 604, 561, 502, 467, 436, 409

δ_H/ppm (400 MHz, $CDCl_3$): 10.42 (1 H, s, (C=O)-H), 8.08 (2 H, d, J 8.3 Hz, Ar-H), 7.91 (1 H, d, J 7.9 Hz, Ar-H), 7.33 (2 H, d, J 8.3 Hz, Ar-H), 6.90 (2 H, m, Ar-H), 3.94 (3 H, s, O-$\underline{CH_3}$), 2.46 (3 H, s, Ar-$\underline{CH_3}$)

δ_C/ppm (100 MHz, $CDCl_3$): 188.80, 164.64, 162.98, 157.27, 145.13, 130.44, 130.06, 129.58, 126.29, 122.79, 114.47, 105.90, 56.06, 21.95

4.2 4-Formyl-3-methoxyphenyl 4-methoxy-2-methylbenzoate

White Solid. Yield: 4.05 g, 74.5 %. RF: 0.49 (40 % ethyl acetate: 60 % 40:60 petroleum ether). M.P = 120 °C

ν_{max}/cm^{-1}: 2931, 1731, 1672, 1605, 1563, 1487, 1473, 1454, 1418, 1404, 1310, 1294, 1236, 1205, 1149, 1122, 1106, 1055, 1022, 947, 882, 821, 795, 761, 734, 691, 661, 640, 611, 580, 556, 517, 480, 468, 453, 402

δ_H/ppm (400 MHz, $CDCl_3$): 10.42 (1 H, s, (C=O)-$\underline{H}$), 8.16 (1 H, d, J 9.2 Hz, Ar-H), 7.90 (1 H, d, J 9.0 Hz, Ar-H), 6.86 (4 H, m, Ar-H), 3.94 (3 H, s, O-$\underline{CH_3}$), 3.88 (3 H, s, O-$\underline{CH_3}$), 2.67 (3 H, s, Ar-$\underline{CH_3}$)

δ_C/ppm (100 MHz, $CDCl_3$): 188.71, 164.36, 163.27, 162.88, 157.29, 144.80, 133.78, 129.88, 122.52, 119.84, 117.32, 114.57, 111.36, 105.90, 55.92, 55.44, 22.59

4.3 4-Formyl-3-methoxyphenyl 2-methoxy-4-methylbenzoate

White Solid. Yield: 4.22 g, 77.6 %. RF: 0.45 (40 % ethyl acetate: 60 % 40:60 petroleum ether). M.P = 140 °C

v_{max}/cm^{-1}: 2864, 1740, 1682, 1608, 1504, 1493, 1458, 1417, 1395, 1292, 1272, 1219, 1187, 1159, 1100, 1020, 931, 871, 834, 817, 799, 768, 745, 722, 694, 670, 631, 608, 550, 529, 484, 466

δ_H/ppm (400 MHz, $CDCl_3$): 10.41 (1 H, s, (C=O)-H), 7.94 (1 H, d, J 7.8 Hz, Ar-H), 7.88 (1 H, d, J 8.4 Hz, Ar-H), 6.88 (4 H, m, Ar-H), 3.93 (3 H, s, O-$\underline{CH_3}$), 3.92 (3 H, s, O-$\underline{CH_3}$), 2.43 (3 H, s, Ar-$\underline{CH_3}$)

δ_C/ppm (100 MHz, $CDCl_3$): 188.75, 163.20, 162.81, 160.45, 157.30, 146.33, 132.52, 129.79, 122.45, 121.17, 115.11, 114.46, 113.00, 105.91, 56.01, 55.91, 22.14

4.4 4-Formyl-3-methoxyphenyl 2,4-dimethylbenzoate

Peach Solid. Yield: 4.88 g, 85.8 %. RF: 0.64 (40 % ethyl acetate: 60 % 40:60 petroleum ether). M.P = 111 °C

v_{max}/cm^{-1}: 1732, 1673, 1604, 1580, 1489, 1454, 1416, 1397, 1308, 1257, 1245, 1226, 1187, 1145, 1106, 1033, 1019, 948, 925, 876, 850, 812, 795, 766, 734, 691, 661, 635, 600, 577, 543, 498, 466, 442

δ_H/ppm (400 MHz, $CDCl_3$): 10.42 (1 H, s, (C=O)-H), 8.07 (1 H, d, J 8.4 Hz, Ar-H), 7.91 (1 H, d, J 9.0 Hz, Ar-H), 7.14 (2 H, m, Ar-H), 6.89 (2 H, m, Ar-H), 3.94 (3 H, s, O-$\underline{CH_3}$), 2.64 (3 H, s, Ar-$\underline{CH_3}$), 2.41 (3 H, s, Ar-$\underline{CH_3}$)

δ_C/ppm (100 MHz, $CDCl_3$): 188.68, 164.82, 162.89, 157.21, 144.05, 141.91, 132.95, 131.50, 129.89, 126.80, 124.83, 122.58, 114.51, 105.86, 55.93, 22.02, 21.55

Compound 5

To a pre-dried flask flushed with argon, **Compound 4** (1 eq) and resorcinol (1.5 eq) were solubilised in DMSO (100 mL for **compound 4.1** or 120 mL for **compounds 4.3 and 4.4**) or tetrahydrofuran (80 mL) and *N,N'*-dimethylformamide (60 mL) for **compound 4.2**. Sodium chlorite (4 eq) and sodium hydrogen phosphate monohydrate (3.5 eq) were solubilised in water (80 mL) before being slowly added to into the reaction flask and the resultant mixture was stirred at room temperature overnight. The quantities of the reagents used in each reaction are listed in **Table S5**. The extent of the reaction was monitored by TLC using an appropriate solvent system (RF values quoted in the product data). The reaction mixture was diluted with water (300 mL) and the pH of the mixture was adjusted to 1 using 32% hydrochloric acid (25 mL). A white solid precipitated after acidification which was collected by vacuum filtration and recrystallised from hot ethanol (150 mL).

Table S5. Quantities of reagents used in the syntheses of the **Compound 5**

Product	Compound 4	Sodium Chlorite	Sodium Hydrogen Phosphate Monohydrate	Resorcinol
5.1	3.10 g, 0.0115 mol	4.16 g, 0.0460 mol	5.56 g, 0.0403 mol	1.90 g, 0.0173 mol
5.2	3.70 g, 0.0123 mol	4.46 g, 0.0493 mol	5.95 g, 0.0431 mol	2.04 g, 0.0185 mol

5.3	4.00 g, 0.0133 mol	4.82 g, 0.0533 mol	6.43 g, 0.0466 mol	2.20 g, 0.0200 mol
5.4	4.50 g, 0.0158 mol	5.72 g, 0.0633 mol	7.63 g, 0.0553 mol	2.61 g, 0.0237 mol

5.1 4-((4-Methylbenzoyl)oxy)-2-methoxybenzoic acid

Yield: 2.54 g, 77.2 %. RF: 0.08 (40 % ethyl acetate:60 % 40:60 petroleum ether). M.P = 204 °C

v_{max}/cm^{-1}: 2816, 1725, 1685, 1672, 1604, 1583, 1499, 1468, 1408, 1303, 1241, 1194, 1177, 1160, 1140, 1095, 1060, 1029, 1018, 947, 892, 838, 787, 770, 747, 668, 652, 593, 554, 481, 445

δ_H/ppm (400 MHz, DMSO-d_6): 12.67 (1 H, s, OH), 8.03 (2 H, d, J 8.0 Hz, Ar-H), 7.75 (1 H, d, J 8.4 Hz, Ar-H), 7.42 (2 H, d, J 8.0 Hz, Ar-H), 7.11 (1 H, d, J 2.0 Hz, Ar-H), 6.92 (1 H, dd, J 8.4 Hz, 2.0 Hz, Ar-H), 3.82 (3 H, s, O CH3), 2.43 (3 H, s, Ar CH3)

δ_C/ppm (100 MHz, DMSO-d_6): 166.58, 164.16, 159.45, 154.32, 144.72, 131.87, 129.93, 129.56, 125.93, 118.70, 113.58, 106.82, 56.13, 21.28

5.2 4-((4-Methoxy-2-methylbenzoyl)oxy)-2-methoxybenzoic acid

Yield: 2.51 g, 64.5 %. RF: 0.06 (40 % ethyl acetate: 60 % 40:60 petroleum ether). M.P = 183 °C

v_{max}/cm^{-1}: 2973, 1722, 1693, 1667, 1606, 1580, 1564, 1503, 1456, 1433, 1401, 1302, 1244, 1189, 1160, 1130, 1091, 1057, 1025, 951, 884, 865, 827, 812, 791, 768, 739, 639, 657, 632, 606, 558, 512, 446

δ_H/ppm (400 MHz, DMSO-d_6): 12.62 (1 H, br, (C=O)-H), 8.11 (1 H, d, J 8.5 Hz, Ar-H), 7.74 (1 H, d, 8.4 Hz, Ar-H), 7.08 (1 H, d, J 2.0 Hz, Ar-H), 6.93 (3 H, m, Ar-H), 3.85 (3 H, s, O-CH_3), 3.82 (3 H, s, O-CH_3), 2.59 (3 H, s, Ar-CH_3)

δ_C/ppm (100 MHz, DMSO-d_6): 167.07, 164.59, 163.26, 159.93, 154.91, 144.02, 133.97, 132.29, 120.18, 118.89, 117.57, 114.24, 112.14, 107.39, 56.57, 55.97, 22.31

5.3 4-((4-Methoxy-2-methylbenzoyl)oxy)-2-methoxybenzoic acid

Yield: 3.10 g, 73.7 %. RF: 0.03 (40 % ethyl acetate: 60 % 40:60 petroleum ether). M.P = 161 °C

v_{max}/cm^{-1}: 2953, 1706, 1664, 1605, 1581, 1500, 1466, 1434, 1405, 1294, 1227, 1190, 1175, 1159, 1133, 1091, 1059, 1028, 954, 902, 829, 781, 769, 720, 694, 660, 608, 547, 516, 445

δ_H/ppm (400 MHz, DMSO-d_6): 12.64 (1 H, br, (C=O)-H), 7.86 (1 H, d, J 7.9 Hz, Ar-H), 7.74 (1 H, d, 8.4 Hz, Ar-H), 7.04 (2 H, m, Ar-H), 6.88 (2 H, m, Ar-H), 3.86 (3 H, s, O-CH_3), 3.82 (3 H, s, O-CH_3), 2.40 (3 H, s, Ar-CH_3)

δ_C/ppm (100 MHz, DMSO-d_6): 167.05, 163.50, 159.92, 154.85, 146.25, 132.32, 121.38, 118.91, 115.52, 114.14, 113.82, 107.25, 56.57, 56.32, 22.02

5.4 4-((2,4-Dimethylbenzoyl)oxy)-2-methoxybenzoic acid

Yield: 3.67 g, 77.3 %. RF: 0.05 (40 % ethyl acetate: 60 % 40:60 petroleum ether). M.P = 162 °C

v_{max}/cm^{-1}: 2842, 1726, 1692, 1667, 1606, 1585, 1499, 1466, 1436, 1404, 1382, 1303, 1268, 1244, 1230, 1188, 1158, 1144, 1132, 1102, 1024, 972, 948, 884, 829, 793, 772, 765, 739, 723, 694, 653, 592, 564, 547, 453, 441

δ_H/ppm (400 MHz, DMSO-d_6): 12.65 (1 H, br, (C=O)-H), 8.01 (1 H, d, J 7.8 Hz, Ar-H), 7.75 (1 H, d, J 8.4 Hz, Ar-H), 7.22 (2 H, m, Ar-H), 7.10 (1 H, d, J 2.0 Hz, Ar-H), 6.91 (1 H, dd, J 8.4 Hz, 2.0 Hz, Ar-H), 3.82 (3 H, s, O-CH_3), 2.56 (3 H, s, Ar-CH_3), 2.37 (3 H, s, Ar-CH_3)

δ_C/ppm (100 MHz, DMSO-d_6): 167.06, 165.09, 159.94, 154.81, 143.98, 141.00, 133.04, 132.32, 131.62, 127.30, 125.47, 119.01, 114.18, 107.35, 56.59, 21.79, 21.48

Compound 6

To a pre-dried flask flushed with argon, **Compound 5** (1 eq), 4-nitrophenol (1.2 eq or 1.5 eq for **compound 5.2**) or 3-fluoro-4-nitrophenol (1.2 eq), and *N,N'*-dicyclohexylcarbodiimide (1.5 eq) were added to the flask. The solids were solubilised with dichloromethane (30 mL) and stirred for 30 min before 4-dimethylaminopyridine (0.15 eq) was added. The quantities of the reagents used in each reaction are listed in **Table S6**. The temperature of the reaction mixture was increased to room temperature and the reaction was allowed to proceed overnight. The white precipitate which formed was removed by vacuum filtration and the filtrate collected. The solvent was removed under vacuum and the crude product was purified using a silica gel column with an appropriate solvent system (RF values quoted in product data). The eluent fractions of interest were evaporated under vacuum to leave a white solid which was recrystallised from hot ethanol (80 mL).

Table S6. Quantities of reagents used in the syntheses of the **Compound 6**

Product	Compound 5	4-Nitrophenol/*3-Fluoro-4-nitrophenol	*N,N'*-Dicyclohexylcarbodiimide	4-Dimethylaminopyridine
6.1	0.300 g, 1.06×10^{-3} mol	0.175 g, 1.26×10^{-3} mol	0.326 g, 1.58×10^{-3} mol	0.019 g, 1.58×10^{-4} mol
6.2	0.300 g, 9.48×10^{-4}	0.198 g, 1.42×10^{-3} mol	0.292 g, 1.42×10^{-3} mol	0.017 g, 1.42×10^{-4} mol
6.3	0.300 g, 9.48×10^{-4}	0.159 g, 1.14×10^{-3} mol	0.292 g, 1.42×10^{-3} mol	0.017 g, 1.42×10^{-4} mol
6.4	0.300 g, 9.99×10^{-4}	0.167 g, 1.20×10^{-3} mol	0.309 g, 1.50×10^{-3} mol	0.018 g, 1.50×10^{-4} mol
6.5	0.300 g, 1.06×10^{-3} mol	*0.198 g, 1.26×10^{-3} mol	0.326 g, 1.58×10^{-3} mol	0.019 g, 1.58×10^{-4} mol
6.6	0.300 g, 9.48×10^{-4}	*0.179 g, 1.14×10^{-3} mol	0.292 g, 1.42x10-3 mol	0.017 g, 1.42×10^{-4} mol
6.7	0.300 g, 9.48×10^{-4}	*0.179 g, 1.14×10^{-3} mol	0.292 g, 1.42x10-3 mol	0.017 g, 1.42×10^{-4} mol
6.8	0.300 g, 9.99×10^{-4}	*0.189 g, 1.20×10^{-3} mol	0.309 g, 1.50×10^{-3} mol	0.018 g, 1.50×10^{-4} mol

6.1 3-Methoxy-4-((4-nitrophenoxy)carbonyl)phenyl 4-methylbenzoate

Yield: 0.077 g, 17.8 %. RF: 0.15 (100 % dichloromethane).

T_{CrN} 164 °C T_{N_FN} (153 °C) T_{NI} 171 °C

v_{max}/cm^{-1}: 1727, 1708, 1606, 1583, 1518, 1492, 1473, 1411, 1344, 1275, 1252, 1237, 1195, 1176, 1160, 1112, 1075, 1053, 1019, 950, 890, 864, 835, 790, 762, 745, 692, 668, 630, 595, 570, 531, 501, 474, 415

δ_H/ppm (400 MHz, DMSO-d_6): 8.32 (2 H, d, J 9.1 Hz, Ar-H), 8.14 (1 H, d, J 9.1 Hz, Ar-H), 8.10 (2 H, d, J 8.1 Hz, Ar-H), 7.42 (2 H, d, J 9.1 Hz, Ar-H), 7.34 (2 H, d, J 8.1 Hz, Ar-H), 6.96 (2 H, m, Ar-H), 3.97 (3 H, s, O-$\underline{CH_3}$), 2.48 (3 H, s, Ar-$\underline{CH_3}$)

δ_C/ppm (100 MHz, $CDCl_3$): 164.51, 162.40, 161.69, 156.52, 155.80, 145.29, 145.07, 133.75, 130.34, 129.48, 126.11, 125.19, 122.75, 115.07, 113.84, 106.43, 56.36, 21.84

MS = $[2M+Na]^+$: Calculated for $C_{44}H_{34}N_2O_{14}Na$: 837.1908. Found: 837.1891. Difference: 2.0 ppm

6.2 3-Methoxy-4-((4-nitrophenoxy)carbonyl)phenyl 4-methoxy-2-methylbenzoate

Yield: 0.065 g, 15.6 %. RF: 0.11 (15 % ethyl acetate: 85 % 40:60 petroleum ether).

T_{CrI} 153 °C T_{N_FN} (113 °C) T_{NI} (120 °C)

v_{max}/cm^{-1}: 2974, 1744, 1728, 1604, 1575, 1519, 1505, 1492, 1454, 1407, 1350, 1329, 1261, 1234, 1191, 1177, 1159, 1121, 1035, 1019, 1001, 935, 886, 861, 830, 812, 772, 764, 742, 720, 691, 670, 647, 614, 592, 543, 496, 455, 415

δ_H/ppm (400 MHz, DMSO-d_6): 8.36 (2 H, d, J 8.7 Hz, Ar-H), 8.15 (1 H, d, J 8.6 Hz, Ar-H), 8.09 (1 H, d, J 8.5 Hz, Ar-H), 7.50 (2 H, d, J 8.7 Hz, Ar-H), 7.24 (1 H, d, J 2.0 Hz, Ar-H), 7.02 (3 H, m, Ar-H), 3.90 (3 H, s, O-$\underline{CH_3}$), 3.86 (3 H, s, O-$\underline{CH_3}$), 2.61 (3 H, s, Ar-$\underline{CH_3}$)

δ_C/ppm (100 MHz, DMSO-d_6): 164.40, 163.36, 162.71, 161.28, 156.58, 156.00, 145.53, 144.17, 134.07, 133.58, 125.77, 123.87, 120.01, 117.61, 115.32, 114.74, 112.20, 107.86, 56.96, 56.01, 22.33

MS = $[M+H]^+$: Calculated for $C_{23}H_{19}NO_8Na$: 460.1008. Found: 460.1031. Difference: 5.0 ppm

6.3 3-Methoxy-4-((4-nitrophenoxy)carbonyl)phenyl 2-methoxy-4-methylbenzoate

Yield: 0.139 g, 33.5 %. RF: 0.28 (100 % dichloromethane).

T_{CrI} 147 °C T_{N_FI} (106 °C)

v_{max}/cm^{-1}: 2920, 1752, 1715, 1611, 1583, 1516, 1489, 1469, 1453, 1410, 1372, 1351, 1285, 1267, 1230, 1203, 1180, 1163, 1154, 1126, 1113, 1084, 1048, 1021, 1007, 931, 882, 863, 822, 764, 743, 723, 692, 671, 623, 608, 547, 499, 462, 417

δ_H/ppm (400 MHz, $CDCl_3$): 8.31 (2 H, d, J 9.2 Hz, Ar-H), 8.12 (1 H, d, J 8.5 Hz, Ar-H), 7.97 (1 H, d, J 7.8 Hz, Ar-H), 7.42 (2 H, d, J 9.2 Hz, Ar-H), 6.96 (2 H, m, Ar-H), 6.88 (2 H, m, Ar-H), 3.95 (6 H, s, O-$\underline{CH_3}$, O-$\underline{CH_3}$), 2.45 (3 H, s, Ar-$\underline{CH_3}$)

δ_C/ppm (100 MHz, $CDCl_3$): 163.16, 162.45, 161.67, 160.51, 156.67, 155.84, 146.43, 145.26, 133.64, 132.55, 125.17, 122.77, 121.19, 115.02, 114.74, 113.97, 113.01, 106.58, 56.34, 56.04, 22.16

MS = $[M+H]^+$: Calculated for $C_{23}H_{19}NO_8Na$: 460.1008. Found: 460.1026. Difference: 1.3 ppm

6.4 3-Methoxy-4-((4-nitrophenoxy)carbonyl)phenyl 2,4-dimethylbenzoate

Yield: 0.160 g, 38.0 %. RF: 0.21 (100 % dichloromethane).

T_{CrI} 150 °C T_{N_FI} (117 °C)

v_{max}/cm^{-1}: 1733, 1706, 1612, 1585, 1520, 1493, 1474, 1412, 1345, 1278, 1263, 1229, 1194, 1160, 1144, 1112, 1069, 1032, 1022, 965, 929, 897, 884, 863, 826, 758, 746, 725, 694, 687, 667, 632, 596, 541, 501, 478, 440, 417

δ_H/ppm (400 MHz, $CDCl_3$): 8.32 (2 H, d, J 8.9 Hz, Ar-H), 8.14 (1 H, d, J 9.1 Hz, Ar-H), 8.09 (1 H, d, J 8.4 Hz, Ar-H), 7.42 (2 H, d, J 8.9 Hz, Ar-H), 7.15 (2 H, m, Ar-H), 6.95 (2 H, m, Ar-H), 3.97 (3 H, s, O-$\underline{CH_3}$), 2.66 (3 H, s, Ar-$\underline{CH_3}$), 2.45 (3 H, s, Ar-$\underline{CH_3}$)

δ_C/ppm (100 MHz, $CDCl_3$): 164.79, 162.42, 161.72, 156.58, 155.81, 145.28, 144.13, 141.97, 133.72, 132.99, 131.52, 126.82, 125.19, 124.77, 122.76, 114.95, 114.00, 106.53, 56.35, 22.04, 21.56

MS = $[2M+Na]^+$: Calculated for $C_{46}H_{38}N_2O_{14}Na$: 865.2221. Found: 865.2231. Difference: 1.2 ppm

6.5 3-Methoxy-4-((3-fluoro-4-nitrophenoxy)carbonyl)phenyl 4-methylbenzoate

Yield: 0.111 g, 24.6 %. RF: 0.22 (100 % dichloromethane).

T_{CrI} 169 °C T_{N_FI} (156 °C)

v_{max}/cm^{-1}: 1754, 1738, 1731, 1610, 1581, 1529, 1484, 1457, 1412, 1347, 1316, 1263, 1219, 1194, 1180, 1168, 1134, 1092, 1069, 1033, 1007, 966, 908, 889, 878, 856, 837, 811, 793, 758, 744, 734, 684, 637, 614, 556, 529, 476, 455

δ_H/ppm (400 MHz, $CDCl_3$): 8.14 (4 H, m, Ar-H), 7.34 (2 H, d, J 8.2 Hz, Ar-H), 7.29 (1 H, dd, J 11.4 Hz, 2.4 Hz, Ar-H), 7.21 (1 H, m, Ar-H), 6.97 (2 H, m, Ar-H), 3.97 (3 H, s, O-$\underline{CH_3}$), 2.48 (3 H, s, Ar-$\underline{CH_3}$)

δ_F/ppm (376 MHz, $CDCl_3$): -113.37 (1 F, s, Ar-F)

δ_C/ppm (100 MHz, $CDCl_3$): 164.47, 161.86, 161.81, 156.74, 156.22 (d, J =266.5 Hz), 155.92 (d, J 10.6 Hz), 145.12, 134.67 (d, J 6.9 Hz), 133.80, 130.34, 129.49, 127.12 (d, J 2.0 Hz), 126.06, 118.20 (d, J 4.0 Hz), 114.57, 113.90, 112.45 (d, J 23.8 Hz), 106.47, 56.37, 21.84.

MS = $[M+Na]^+$: Calculated for $C_{22}H_{16}NO_7FNa$: 448.0808. Found: 448.0814. Difference: 1.3 ppm

6.6 3-Methoxy-4-((3-fluoro-4-nitrophenoxy)carbonyl)phenyl 4-methoxy-2-methylbenzoate

Yield: 0.110 g, 25.4 %. RF: 0.23 (100 % dichloromethane).

T_{CrI} 139 °C T_{N_FI} (109 °C)

v_{max}/cm^{-1}: 1756, 1732, 1717, 1606, 1574, 1521, 1505, 1455, 1409, 1347, 1331, 1282, 1227, 1189, 1165, 1154, 1122, 1093, 1064, 1029, 1009, 973, 935, 879, 865, 847, 817, 760, 744, 719, 683, 671, 632, 613, 592, 558, 545, 521, 446, 414

δ_H/ppm (400 MHz, $CDCl_3$): 8.18 (2 H, m, Ar-H), 8.11 (1 H, d, J 9.1 Hz, Ar-H), 7.28 (1 H, dd, J 11.4 Hz, 2.4 Hz, Ar-H), 7.21 (1 H, ddd, J 9.1 Hz, 2.4 Hz, 1.3 Hz, Ar-H), 6.94 (2 H, m, Ar-H), 6.84 (2 H, m, Ar-H), 3.97 (3 H, s, O-$\underline{CH_3}$), 3.89 (3 H, s, O-$\underline{CH_3}$), 2.68 (3 H, s, Ar-$\underline{CH_3}$)

δ_F/ppm (376 MHz, $CDCl_3$): -113.39 (1 F, s, Ar-F)

δ_C/ppm (100 MHz, $CDCl_3$): 164.27, 163.34, 161.89, 161.85, 156.90, 156.22 (d, J 266.5 Hz), 155.96 (d, J 10.6 Hz), 144.88, 134.64 (d, J 6.9 Hz), 133.80, 133.75, 127.10 (d, J 2.2 Hz), 119.72, 118.21 (d, J 4.0 Hz), 117.34, 114.34, 114.12, 112.45 (d, J 23.8 Hz), 111.41, 106.61, 56.35, 55.46, 22.61.

MS = $[M+Na]^+$: Calculated for $C_{23}H_{18}NO_8FNa$: 478.0914. Found: 478.0913. Difference: 0.2 ppm

6.7 3-Methoxy-4-((3-fluoro-4-nitrophenoxy)carbonyl)phenyl 2-methoxy-4-methylbenzoate

Yield: 0.100 g, 23.2 %. RF: 0.30 (100 % dichloromethane).

T_{CrI} 171 °C T_{N_FI} (106 °C)

ν_{max}/cm^{-1}: 2948, 1733, 1605, 1581, 1524, 1487, 1466, 1450, 1409, 1353, 1290, 1266, 1213, 1192, 1156, 1132, 1093, 1029, 1012, 969, 927, 894, 864, 842, 833, 809, 769, 759, 722, 686, 671, 564, 548, 531, 518, 459

δ_H/ppm (400 MHz, $CDCl_3$): 8.17 (1 H, dd, J 8.9 Hz, 8.6 Hz, Ar-H), 8.10 (1 H, d, J 8.6 Hz, Ar-H), 7.97 (1 H, d, J 7.9 Hz, Ar-H), 7.21 (1 H, m, Ar-H), 6.96 (2 H, m, Ar-H), 6.88 (2 H, m, Ar-H), 3.96 (3 H, s, O-$\underline{CH_3}$), 3.95 (3 H, s, O-$\underline{CH_3}$), 2.45 (3 H, s, Ar-$\underline{CH_3}$)

δ_F/ppm (376 MHz, $CDCl_3$): -113.41 (1 F, s, Ar-F),

δ_C/ppm (100 MHz, $CDCl_3$): 163.11, 161.90, 161.80, 160.53, 156.90, 156.22 (d, J 266.5 Hz), 155.97 (d, J 10.5 Hz), 146.48, 134.63 (d, J 7.5 Hz), 133.69, 132.56, 127.10 (d, J 2.0 Hz), 121.19, 118.22 (d, J 3.9 Hz), 114.96, 114.24, 114.03, 113.01, 112.46 (d, J 23.7 Hz), 106.61, 56.35, 56.04, 22.17.

MS = $[M+H]^+$: Calculated for $C_{23}H_{18}NO_8FNa$: 478.0914. Found: 478.0893. Difference: 4.4 ppm

6.8 3-Methoxy-4-((3-fluoro-4-nitrophenoxy)carbonyl)phenyl 2,4-dimethylbenzoate

Yield: 0.147 g, 33.5 %. RF: 0.37 (100 % dichloromethane).

T_{CrI} 136 °C T_{N_FI} (116 °C)

ν_{max}/cm^{-1}: 2924, 1749, 1729, 1717, 1605, 1585, 1526, 1495, 1471, 1454, 1412, 1344, 1281, 1222, 1190, 1152, 1143, 1115, 1093, 1066, 1022, 970, 930, 888, 873, 844, 760, 746, 724, 685, 670, 632, 609, 547, 518, 462, 438, 417

δ_H/ppm (400 MHz, $CDCl_3$): 8.14 (3 H, m, Ar-H), 7.29 (1 H, dd, J 11.4 Hz, 2.4 Hz, Ar-H), 7.17 (3 H, m, Ar-H), 6.95 (2 H, m, Ar-H), 3.97 (3 H, s, O-$\underline{CH_3}$), 2.66 (3 H, s, Ar-$\underline{CH_3}$), 2.42 (3 H, s, Ar-$\underline{CH_3}$)

δ_F/ppm (376 MHz, $CDCl_3$): -113.42 (1 F, s, Ar-F)

δ_C/ppm (100 MHz, $CDCl_3$): 164.74, 161.88, 161.85, 156.81, 156.22 (d, J 266.6 Hz), 155.94 (d, J 10.6 Hz), 144.18, 142.00, 134.66 (d, J 7.3 Hz), 133.77, 133.00, 131.52, 127.11 (d, J 2.1 Hz), 126.83, 124.71, 118.20 (d, J 4.0 Hz), 114.44, 114.06, 112.45 (d, J 23.8 Hz), 106.56, 56.36, 22.04, 21.57

MS = $[2M+Na]^+$: Calculated for $C_{46}H_{36}N_2O_{14}F_2Na$: 901.2032. Found: 901.2011. Difference: 2.3 ppm
